# Assessing the Accuracy of Responses by the Language Model ChatGPT to Questions Regarding Bariatric Surgery

**DOI:** 10.1007/s11695-023-06603-5

**Published:** 2023-04-27

**Authors:** Jamil S. Samaan, Yee Hui Yeo, Nithya Rajeev, Lauren Hawley, Stuart Abel, Wee Han Ng, Nitin Srinivasan, Justin Park, Miguel Burch, Rabindra Watson, Omer Liran, Kamran Samakar

**Affiliations:** 1 Karsh Division of Gastroenterology and Hepatology, Cedars-Sinai Medical Center, 8700 Beverly Blvd, Los Angeles, CA 90048 USA; 2 Division of Upper GI and General Surgery, Department of Surgery, Health Care Consultation Center, Keck School of Medicine of USC, 1510 San Pablo St. #514, Los Angeles, CA 90033 USA; 3 Bristol Medical School, University of Bristol, 5 Tyndall Ave, Bristol, BS8 1UD UK; 4 Department of Surgery, Cedars-Sinai Medical Center, 8700 Beverly Blvd, Los Angeles, CA 90048 USA; 5 Department of Psychiatry and Behavioral Sciences, Cedars-Sinai Medical Center, 8700 Beverly Blvd, Los Angeles, CA 90048 USA; 6 Division of Health Services Research, Department of Medicine, Cedars-Sinai Medical Center, 8700 Beverly Blvd, Los Angeles, CA 90048 USA

**Keywords:** Artificial intelligence, ChatGPT, Language learning models, Bariatric surgery, Weight loss, Health literacy

## Abstract

**Purpose:**

ChatGPT is a large language model trained on a large dataset covering a broad range of topics, including the medical literature. We aim to examine its accuracy and reproducibility in answering patient questions regarding bariatric surgery.

**Materials and methods:**

Questions were gathered from nationally regarded professional societies and health institutions as well as Facebook support groups. Board-certified bariatric surgeons graded the accuracy and reproducibility of responses. The grading scale included the following: (1) comprehensive, (2) correct but inadequate, (3) some correct and some incorrect, and (4) completely incorrect. Reproducibility was determined by asking the model each question twice and examining difference in grading category between the two responses.

**Results:**

In total, 151 questions related to bariatric surgery were included. The model provided “comprehensive” responses to 131/151 (86.8%) of questions. When examined by category, the model provided “comprehensive” responses to 93.8% of questions related to “efficacy, eligibility and procedure options”; 93.3% related to “preoperative preparation”; 85.3% related to “recovery, risks, and complications”; 88.2% related to “lifestyle changes”; and 66.7% related to “other”. The model provided reproducible answers to 137 (90.7%) of questions.

**Conclusion:**

The large language model ChatGPT often provided accurate and reproducible responses to common questions related to bariatric surgery. ChatGPT may serve as a helpful adjunct information resource for patients regarding bariatric surgery in addition to standard of care provided by licensed healthcare professionals. We encourage future studies to examine how to leverage this disruptive technology to improve patient outcomes and quality of life.

**Graphical Abstract:**

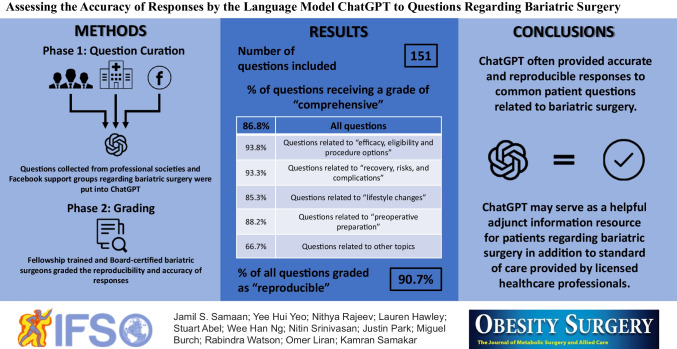

**Supplementary Information:**

The online version contains supplementary material available at 10.1007/s11695-023-06603-5.

## Introduction

The utilization of artificial intelligence (AI) in medicine has grown significantly in recent years with the advent of a broad range of applications and technologies used to improve patient care and evidence-based medicine. These technologies are often used by healthcare providers or the healthcare system for patient care and research. On November 30, 2022, the chatbot ChatGPT was launched by the company OpenAI for public use through a free online platform available to all users with a free account. ChatGPT is an AI large language model trained on a large dataset covering a broad range of topics, including the medical literature. When prompted with inquiries, ChatGPT can provide well-formulated, conversational, and easy-to-understand seemingly knowledgeable responses. Discussion regarding the potential of ChatGPT to disrupt all fields of academia is ongoing, and its applicability is actively under investigation [1, 2]. One recent study-showed ChatGPT achieved a passing score when prompted with USMLE style questions, while others have shown its appropriateness in responding to cardiovascular disease prevention-related inquiries and accuracy in responding to commonly asked cirrhosis and hepatocellular carcinoma-related questions [3-5]. There are currently no studies examining the clinical application of ChatGPT in the field of bariatric surgery.

Obesity rates are on the rise in the USA, with the prevalence of obesity and severe obesity increasing over the past two decades [6]. Bariatric surgery has been shown to be an effective and safe therapeutic modality for long-term weight loss as well as resolution of obesity-related comorbidities such as diabetes mellitus, hypertension, and obstructive sleep apnea [7]. Despite its proven efficacy and safety, less than 1% of eligible patients undergo bariatric surgery in the USA [8]. The reason for this underutilization is likely multifactorial, and multiple studies have highlighted the various barriers to care such as provider perceptions, insurance coverage, access to resources, and patient perceptions [9,10]. Some of these barriers are rooted in the lack of familiarity with the safety and efficacy of bariatric surgery, making it difficult for patients and their providers to make informed decisions regarding referring for bariatric surgery.

Even after obtaining a referral, patients typically undergo a preoperative evaluation process and optimization of preexisting comorbidities, as well as receive education and lifestyle counseling. The literature, while limited, reveals that patients often seek sources of information outside of their healthcare provider’s office, with various social media venues being a popular source of information [11]. In light of this, and given ChatGPTs rapidly growing number of users, we anticipate patients may utilize ChatGPT as a source of information as part of their weight loss journey. Therefore, it is crucial to determine the reliability of this new tool. In this study, we aimed to examine the accuracy and reproducibility of ChatGPT’s responses to commonly asked patient questions regarding bariatric surgery.

## Methods

### Question Curation/Data Source

**Fig. 1:**
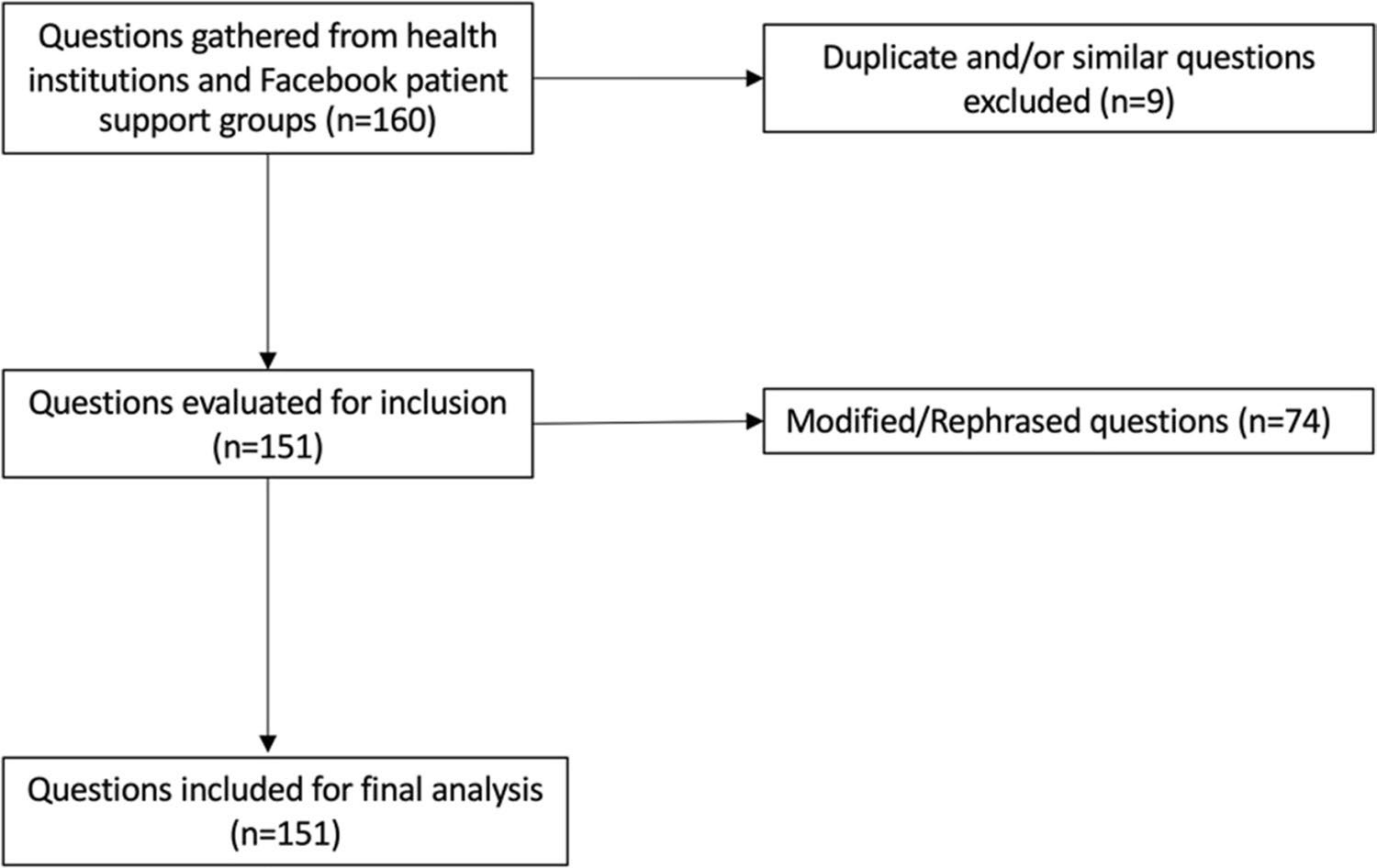
Flow chart of bariatric surgery-related question selection

Questions were obtained from the Frequently Asked Questions pages from the American Society for Metabolic and Bariatric Surgery (ASMBS) and MedlinePlus Medical Encyclopedia, along with two social media support groups for patients who were considering, eligible for, or who had recently undergone a bariatric procedure: Bariatric Surgery & Gastric Sleeve Support Group (Facebook) and Gastric Sleeve and Bariatric Surgery Group, Supporting the New You (Facebook). Questions were curated, screened, and approved by three authors (J. S., N. R., K. S.) to evaluate their inclusion in the study. Only questions regarding bariatric surgery or weight loss surgery were included. Duplicate and similar questions from multiple sources were removed (Fig. 1). Questions requiring subjective or personalized responses (e.g., How will bariatric surgery change my lifespan?) and questions that were vague (e.g., How much weight is normally lost after this surgery?) were rephrased to a generic language format to allow for inclusion in the study (Fig. 1). Other questions were grammatically modified to eliminate ambiguity. A total of 151 questions were included and were used to generate responses from ChatGPT. To better characterize ChatGPTs performance in various topics within bariatric surgery, questions were categorized into multiple groups for statistical analysis purposes: (1) eligibility, efficacy, and procedure options; (2) preoperative preparation; (3) recovery, risks, and complications; (4) lifestyle changes; and (5) other.

### ChatGPT

ChatGPT is a large language model trained on a large database of information from a wide range of sources including online websites, books, and articles leading up to 2021 [12]. When prompted with inquiries, ChatGPT can provide well-formulated, conversational, and easy-to-understand responses. The creators utilized reinforcement learning from human feedback (RLHF) to fine-tune the model to follow a broad class of commands and written instructions fine-tuned by human preferences as a reward signal [13]. The model was also trained to align with user intentions and minimize bias, toxic, or otherwise harmful responses. The source of information used to train ChatGPT is unknown.

### Response Generation

To generate responses, each question was prompted to ChatGPT (December 15th version). Each individual question was inputted twice on separate occasions using the “new chat” function in order to generate two responses per question. This was done to determine the reproducibility of responses to the same question.

### Question Grading

Responses to questions were first independently graded for accuracy and reproducibility by two board-certified, fellowship-trained, active practice academic bariatric surgeon reviewers (L. H. and S. A.). Reviewers were instructed to grade the accuracy of responses based on known information leading up to 2021. Reproducibility was graded based on the similarity in accuracy of the two responses per question generated by ChatGPT. If the responses were similar, only the first response from ChatGPT was graded. If the responses were not similar, both responses were individually graded.

Accuracy of each response was graded with the following scale:

1. Comprehensive: Defined as accurate and comprehensive, nothing more a board-certified bariatric surgeon can add if asked this question by a patient
2. Correct but inadequate: All information is correct but incomplete; a board-certified bariatric surgeon would have more important information to add if asked this question by a patient.
3. Some correct and some incorrect
4. Completely incorrect

Disagreement in reproducibility or grading of each response was resolved by a third board-certified, fellowship-trained, active practice academic bariatric surgeon (K. S.). The final grades were then compiled and used to analyze the overall performance of ChatGPT in answering questions related to bariatric surgery.

### Statistical Analysis

The proportions of responses earning each grade were calculated. To determine reproducibility, responses were categorized into two groups: a grade of 1 and 2 comprised the first group, and a grade of 3 and 4 comprised the second group. The two responses to each question were considered significantly different from one another, or not reproducible, if the assigned grades for each response fell under different groups. Interrater reliability between reviewer 1 and reviewer 2 was tested using Kappa statistic and showed moderate agreement with a Kappa value of 0.762 with a 95% confidence interval of 0.684–0.825. All analyses were conducted using Microsoft Excel (version 16.69.1).

## Results

In total, 151 questions related to bariatric surgery were inputted into ChatGPT (Supplementary Table 1). The model provided “comprehensive” responses to 131/151 (86.8%) of questions. The model provided “comprehensive” responses to 131/151 (86.8%) of questions. When examined by category, the model provided “comprehensive” responses to 93.8% of questions related to “efficacy, eligibility and procedure options”; 93.3% to “preoperative preparation”; 85.3% to “recovery, risks, and complications”; and 88.2% to “lifestyle changes” (Table 1). The percentage of comprehensive responses was lower for the “others” category, with only two-thirds of responses graded as “comprehensive,” while 25% of responses were graded as “some correct and some incorrect.” In total, 4 questions across all categories were given a rating of “completely incorrect.” For example, when prompted regarding the expected scars from surgery, the model described the open approach rather than the commonly used laparoscopic approach. When prompted regarding the use of straws after surgery, the model explained “Using a straw can help to bypass the smaller stomach and deliver fluids directly to the intestines, where they can be absorbed more easily.”

**Table 1:** Grading of responses generated by ChatGPT to questions related to bariatric surgery categorized by question type

Eligibility, efficacy, and procedure options (*N* = 32)	
1. Comprehensive	93.8%
2. Correct but incomplete	3.1%
3. Some correct and some incorrect	0.0%
4. Completely incorrect	3.1%
Preoperative preparation (*N* = 15)	
1. Comprehensive	93.3%
2. Correct but incomplete	0.0%
3. Some correct and some incorrect	6.7%
4. Completely incorrect	0.0%
Recovery, risks, and complications (*N* = 75)	
1. Comprehensive	85.3%
2. Correct but incomplete	6.7%
3. Some correct and some incorrect	4.0%
4. Completely incorrect	4.0%
Lifestyle changes (*N* = 17)	
1. Comprehensive	88.2%
2. Correct but incomplete	5.9%
3. Some correct and some incorrect	5.9%
4. Completely incorrect	0.0%
Others (*N* = 12)	
1. Comprehensive	66.7%
2. Correct but incomplete	8.3%
3. Some correct and some incorrect	25.0%
4. Completely incorrect	0.0%

The model provided reproducible answers to 137 (90.7%) of questions. Reproducibility of responses in the “lifestyle changes” and “others” categories was 100% but lower in the “recovery, risks and complications” (86.7%); “eligibility, efficacy and procedure options” (90.6%); and “preoperative preparation” (93.3%) categories (Table 2).

**Table 2:** Proportion of questions with reproducible responses generated by ChatGPT categorized by question type

Eligibility, efficacy, and procedure options (*N* = 32)	90.6%
Preoperative preparation (*N* = 15)	93.3%
Recovery, risks, and complications (*N* = 75)	86.7%
Lifestyle changes after surgery (*N* = 17)	100%
Others (*N* = 12)	100%

Reproducibility was defined as no difference in grading categories (1 and 2 vs 3 and 4) between the two responses for each question

## Discussion

We examined the accuracy and reproducibility of the new AI model ChatGPT in responding to commonly asked patient questions related to bariatric surgery. The responses of ChatGPT to patient questions obtained from nationally regarded professional societies and health institutions as well as Facebook patient support groups were independently assessed for accuracy and reproducibility by a panel of board-certified and fellowship-trained bariatric surgeons. Overall, ChatGPT’s responses were accurate with most responses graded as “comprehensive” on our grading scale along with high reproducibility. The model performed well in the majority of topics such as “efficacy, eligibility and procedure options”; “preoperative preparation”; “recovery, risks, and complications”; and “lifestyle changes.” Our study shows the potential significant impact of this technology in improving patients’ experience and outcomes in the future. Furthermore, we highlight important limitations and considerations.

Previous literature has shown patients seek information related to bariatric surgery outside of their healthcare providers, with social media serving as a popular resource [11, 14, 15]. Multiple studies have shown limitations in the quality and reliability of online sources of information regarding bariatric surgery, including social media [16-18]. Furthermore, research has demonstrated that there is a lack of easy-to-understand online resources about bariatric surgery with over 93% of websites surveyed receiving a rating of unacceptable readability [19]. Furthermore, navigating current search engines can be difficult when searching for specific answers. Search results may be overwhelming, not relevant to the question asked or difficult to ascertain the reliability and accuracy of sources. Given these limitations, we hypothesize that patients will utilize ChatGPT as a source of information for their bariatric surgery-related questions. Our reviewers found ChatGPT easy to access and understand. Universal access AI models may also serve to bridge access and knowledge disparities in communities with limited resources. Our study findings are encouraging, and we anticipate significant future interest in this technology as it relates not only to bariatric surgery but also other fields of medicine.

ChatGPT has the potential to mitigate disparities in health literacy among bariatric surgery candidates and patients. Levels of health literacy have been shown to impact bariatric surgery outcomes with lower literacy being associated with lower short-term as well as long-term postoperative weight loss [20-22]. Other studies have shown the association of health literacy with adherence to medical appointments 1 year after bariatric surgery [23]. One study demonstrated that low health literacy among bariatric surgery candidates had a negative association with eventually undergoing bariatric surgery [24]. While there are no studies that have explored the impact of health literacy on bariatric surgery complications, health literacy has been shown to be negatively associated with postoperative complications after colorectal surgery [25, 26]. Our study demonstrates that the currently available version of ChatGPT provided comprehensive responses to questions regarding the safety, efficacy, and complications of bariatric surgery for most questions reviewed. Future iterations of this technology will likely offer improvements in accuracy and readability. While there are no current studies examining the impact of the use of ChatGPT on health literacy, we hypothesize that utilization of this model will provide patients with an easy-to-understand, accurate, and reproducible resource of information for patients.

ChatGPT may also serve as a tool for improving the perception of the safety and efficacy of bariatric surgery both among prospective candidates and the general public, thereby increasing its adoption as a treatment modality for severe obesity. Despite its known efficacy, less than 1% of eligible patients undergo bariatric surgery. Previous studies have shown both lack of familiarity and discordance between patient perceptions and the demonstrated clinical safety and efficacy profile of bariatric surgery [10]. These trends were also found among the general public. Furthermore, portrayal of bariatric surgery in the media may also contribute to its underutilization with one study in the UK finding 33% of articles published in newspapers were negatively slanted against bariatric surgery [27]. Having easy access to information regarding bariatric surgery may help raise awareness among the general public and eligible candidates, thereby decreasing the stigma surrounding surgery and potentially increase its utilization.

### Strengths and Limitations

To our knowledge, this is the first study to examine the utility of the model ChatGPT in the field of bariatric surgery. Patient questions were obtained from well-regarded sources and societies as well as patient questions from Facebook support groups to provide a comprehensive and realistic sample of patient questions. Responses were independently graded by board-certified bariatric surgeons with discrepancies resolved by a blinded third senior reviewer to comprehensively evaluate the accuracy and reproducibility of ChatGPTs responses.

There are several limitations of ChatGPT that should be highlighted and considered when evaluating it as a potential source of information for patients. While the model performed well with accurate and often reproducible responses, there were several responses that contained incorrect information which may be dangerous to patients without the direction of a healthcare provider. This limitation supports the potential use of ChatGPT as an adjunct source of information but not a replacement of the standard of care provided by a team of licensed healthcare professionals. We hope this limitation is minimized with the expected continuous improvement of the model overtime and in turn provide more accurate and reproducible responses. Secondly, recommendations and standards of practice may vary by region, medical society, or country of residence, providing a challenge in assessing the accuracy of responses for each user around the country or world regarding certain topics. The source of information in which ChatGPT uses to produce responses is unknown which may impact the reliability of its responses for certain topics. Third, ChatGPT may write linguistically convincing responses which are incorrect or nonsensical. This false sense of confidence in responses may lead to the adoption of incorrect and potentially dangerous recommendations by unsuspecting users. OpenAI reports that the current version of the model is expected to produce false positives and false negatives but hope to minimize these limitations with user feedback and ongoing improvement in the model.

### Future Directions

ChatGPT and future similar technologies are expected to revolutionize all fields of academia and likely the field of medicine, although its utility and implementation process remain unclear. If validated by future studies, ChatGPT can be a powerful tool to empower patients. Its performance in answering patient questions regarding cirrhosis and hepatocellular carcinoma has been studied with promising results, similar to the current study [5]. We encourage future studies to expand on our findings in order to study the utility of this revolutionary technology in improving patient experience and outcomes in bariatric surgery.

## Conclusion

The large language model ChatGPT provided accurate and reproducible responses to common questions related to bariatric surgery. ChatGPT may serve as a helpful adjunct source of information regarding bariatric surgery for patients and surgical candidates in addition to standard of care provided by licensed healthcare professionals. We encourage future studies to examine how to leverage this disruptive technology to improve patient outcomes and quality of life.

## Supplementary Information

ESM 1:

## References

[1] O'Connor S (2023). ChatGPT. Open artificial intelligence platforms in nursing education: tools for academic progress or abuse?. Nurse Educ Pract..

[2] Graham F. Daily briefing: will ChatGPT kill the essay assignment? Nature. 2022; 10.1038/d41586-022-04437-2. 10.1038/d41586-022-04437-2 36517680

[3] Gilson A, Safranek CW, Huang T, Socrates V, Chi L, Taylor RA, Chartash D (2023). How does ChatGPT perform on the United States Medical Licensing Examination? The implications of large language models for medical education and knowledge assessment. JMIR Med Educ..

[4] Sarraju A, Bruemmer D, Van Iterson E, Cho L, Rodriguez F, Laffin L (2023). Appropriateness of cardiovascular disease prevention recommendations obtained from a popular online chat-based artificial intelligence model. JAMA..

[5] Yeo YH, Samaan JS, Ng WH, Ting PS, Trivedi H, Vipani A, Ayoub W, Yang JD, Liran O, Spiegel B, Kuo A. Assessing the performance of ChatGPT in answering questions regarding cirrhosis and hepatocellular carcinoma. Clin Mol Hepatol. 2023; 10.3350/cmh.2023.0089. 10.3350/cmh.2023.0089 PMC10366809 36946005

[6] Obesity is a common, serious, and costly disease. Centers for Disease Control and Prevention. Accessed January 21, 2023. https://www.cdc.gov/obesity/data/adult.html

[7] Arterburn DE, Telem DA, Kushner RF, Courcoulas AP (2020). Benefits and risks of bariatric surgery in adults: a review. JAMA..

[8] New study finds most bariatric surgeries performed in northeast, and fewest in south where obesity rates are highest, and economies are weakest. American Society for Metabolic and Bariatric Surgery. Published online November 15, 2018. Accessed January 23, 2023. https://asmbs.org/articles/new-study-finds-most-bariatric-surgeries-performed-in-northeast-and-fewest-in-south-where-obesity-rates-are-highest-and-economies-are-weakest

[9] Premkumar A, Samaan JS, Samakar K (2022). Factors associated with bariatric surgery referral patterns: a systematic review. J Surg Res..

[10] Rajeev ND, Samaan JS, Premkumar A, Srinivasan N, Yu E, Samakar K (2023). Patient and the public’s perceptions of bariatric surgery: a systematic review. J Surg Res..

[11] Scarano Pereira JP, Martinino A, Manicone F, Scarano Pereira ML, Iglesias Puzas Á, Pouwels S, Martínez JM (2022). Bariatric surgery on social media: a cross-sectional study. Obes Res Clin Pract..

[12] openai. ChatGPT: optimizing language models for dialogue. 2023; https://openai.com/blog/chatgpt/. Accessed 1/1/2023, 2023.

[13] Ouyang L, Wu J, Jiang X (2022). Training language models to follow instructions with human feedback. Adv Neur Inform Proc Syst..

[14] Athanasiadis DI, Roper A, Hilgendorf W, Voss A, Zike T, Embry M, Banerjee A, Selzer D, Stefanidis D (2021). Facebook groups provide effective social support to patients after bariatric surgery. Surg Endosc..

[15] Koball AM, Jester DJ, Domoff SE, Kallies KJ, Grothe KB, Kothari SN (2017). Examination of bariatric surgery Facebook support groups: a content analysis. Surg Obes Relat Dis..

[16] Batar N, Kermen S, Sevdin S, Yıldız N, Güçlü D (2020). Assessment of the quality and reliability of information on nutrition after bariatric surgery on YouTube. Obes Surg..

[17] Corcelles R, Daigle CR, Talamas HR, Brethauer SA, Schauer PR (2015). Assessment of the quality of Internet information on sleeve gastrectomy. Surg Obes Relat Dis..

[18] Koball AM, Jester DJ, Pruitt MA, Cripe RV, Henschied JJ, Domoff S (2018). Content and accuracy of nutrition-related posts in bariatric surgery Facebook support groups. Surg Obes Relat Dis..

[19] Meleo-Erwin Z, Basch C, Fera J, Ethan D, Garcia P (2019). Readability of online patient-based information on bariatric surgery. Health Promot Perspect..

[20] Mahoney ST, Strassle PD, Farrell TM, Duke MC (2019). Does lower level of education and health literacy affect successful outcomes in bariatric surgery?. J Laparoendosc Adv Surg Tech A..

[21] Erdogdu UE, Cayci HM, Tardu A, Demirci H, Kisakol G, Guclu M (2019). Health literacy and weight loss after bariatric surgery. Obes Surg..

[22] Miller-Matero LR, Hecht L, Patel S, Martens KM, Hamann A, Carlin AM (2021). The influence of health literacy and health numeracy on weight loss outcomes following bariatric surgery. Surg Obes Relat Dis..

[23] Hecht LM, Martens KM, Pester BD, Hamann A, Carlin AM, Miller-Matero LR (2022). Adherence to medical appointments among patients undergoing bariatric surgery: do health literacy, health numeracy, and cognitive functioning play a role?. Obes Surg..

[24] Hecht L, Cain S, Clark-Sienkiewicz SM, Martens K, Hamann A, Carlin AM, Miller-Matero LR (2019). Health literacy, health numeracy, and cognitive functioning among bariatric surgery candidates. Obes Surg..

[25] Theiss LM, Wood T, McLeod MC, Shao C, Santos Marques ID, Bajpai S, Lopez E, Duong AM, Hollis R, Morris MS, Chu DI (2022). The association of health literacy and postoperative complications after colorectal surgery: a cohort study. Am J Surg..

[26] Baker S, Malone E, Graham L, Dasinger E, Wahl T, Titan A, Richman J, Copeland L, Burns E, Whittle J, Hawn M, Morris M (2020). Patient-reported health literacy scores are associated with readmissions following surgery. Am J Surg..

[27] Williamson JM, Rink JA, Hewin DH (2012). The portrayal of bariatric surgery in the UK print media. Obes Surg..

