# Supplementary material for: Assessing the Accuracy of Responses by the Language Model ChatGPT to Questions Regarding Bariatric Surgery
Source: Obes Surg. 2023 Apr 27;33(6):1790–6. doi: 10.1007/s11695-023-06603-5 (PMC10234918; doi:10.1007/s11695-023-06603-5)
Supplement: Supplementary file 1 — ESM 1: [file 11695_2023_6603_MOESM1_ESM.docx]

**Supplemental File.**

Assessing the Accuracy of Responses by the Language Model ChatGPT to Questions Regarding Bariatric Surgery

**Table of Contents**

*Supplemental Table 1. Scoring of responses generated from ChatGPT for questions related to bariatric surgery* ***2***

**Supplemental Table 1. Scoring of responses generated from ChatGPT for questions related to bariatric surgery**

| Questions | Response 1 | Response 2 | Reviewer 1 grades 1. Comprehensive  2. Correct but inadequate  3. Some correct and some incorrect 4. Completely incorrect | Reviewer 2 grades 1. Comprehensive  2. Correct but inadequate  3. Some correct and some incorrect 4. Completely incorrect | Reviewer 3 grades for the responses with discrepancies |
| --- | --- | --- | --- | --- | --- |
| Will weight loss surgery cure pseudotumor cerebri syndrome? | Pseudotumor cerebri syndrome (also known as idiopathic intracranial hypertension) is a condition that is characterized by increased pressure in the skull, which can cause symptoms such as headache, vision problems, and ringing in the ears. In some cases, weight loss can help to improve symptoms of pseudotumor cerebri syndrome, but weight loss surgery is generally not considered a cure for the condition.  Weight loss surgery can help to promote significant and sustained weight loss, which can help to reduce the risk of developing or worsening pseudotumor cerebri syndrome. However, the exact relationship between weight loss and pseudotumor cerebri syndrome is not fully understood, and more research is needed to determine the optimal approach for managing the condition.  If you have pseudotumor cerebri syndrome and are considering weight loss surgery, it is important to carefully consider the potential risks and benefits of the procedure with your healthcare team and to discuss your specific goals and expectations. Your healthcare team can provide you with more information about the potential role of weight loss surgery in the management of pseudotumor cerebri syndrome and can help you to explore other treatment options that may be appropriate for you. | Similar | 1 | 1 | N/A |
| Are people who have had an organ transplant before eligible for bariatric surgery? | Eligibility for weight loss surgery, also known as bariatric surgery, is typically determined on a case-by-case basis. People who have had an organ transplant may be eligible for bariatric surgery, but it will depend on their overall health and the specific type of surgery they are considering.  Bariatric surgery is a major procedure that carries risks and requires a lifelong commitment to healthy lifestyle changes, such as following a healthy diet and getting regular exercise. It is generally recommended for people who have a body mass index (BMI) of 40 or higher (severe obesity) or a BMI of 35 or higher (obesity) and who have been unable to achieve significant and sustained weight loss through diet and exercise alone.  If you have had an organ transplant and are considering weight loss surgery, it is important to talk to your healthcare provider. They can evaluate your health and determine if you are a candidate for the surgery. They can also help you understand the potential risks and benefits of the surgery and recommend the best course of action for you. | Similar | 1 | 1 | N/A |
| How long after a heart attack can you have weight loss surgery? | The timing of when you can have weight loss surgery after a heart attack will depend on your individual situation and will be determined by your doctor. Weight loss surgery, such as gastric bypass or sleeve gastrectomy, is a major surgical procedure that carries some risks and potential complications, including bleeding, infection, and complications related to anesthesia.  It is generally recommended to wait at least six to twelve months after a heart attack before having weight loss surgery. This allows time for your heart to fully recover and to adjust to any changes in medication or lifestyle that may have been made after the heart attack. During this time, it is important to focus on improving your overall health and to work with your healthcare team to manage any underlying health conditions that may be contributing to your excess weight.  If you are considering weight loss surgery after a heart attack, it is important to speak with your doctor or a medical professional for guidance. They can evaluate your specific situation and determine if the surgery is appropriate for you and, if so, when it would be safe to have the procedure. | The timing of weight loss surgery after a heart attack depends on several factors, including the severity of the heart attack, the individual's overall health and medical history, and the specific type of weight loss surgery being considered.  In general, it is recommended to wait at least three to six months after a heart attack to allow time for the heart to recover and to ensure that the individual is stable before undergoing a major surgery. However, the specific length of time may vary depending on the individual's circumstances. For example, if the heart attack was severe or if the individual has other underlying health conditions that may increase the risk of complications during surgery, it may be necessary to wait longer before undergoing weight loss surgery.  It is important to discuss the timing of weight loss surgery with your healthcare provider and to carefully consider the potential risks and benefits of the procedure in the context of your overall health. Your healthcare provider can help you to determine the most appropriate time for surgery and can provide guidance on how to prepare for the procedure. | 1, 3 | 1, 2 | 1, 3 |
| Are amputees eligible for weight loss surgery? | Amputees may be eligible for weight loss surgery depending on their individual circumstances. The decision to undergo weight loss surgery is typically based on a person's overall health, body mass index (BMI), and other factors.  If you are an amputee and are considering weight loss surgery, it is important to speak with a healthcare professional, such as a bariatric surgeon or a primary care doctor, to determine if it is a safe and appropriate option for you. They will be able to assess your health and weight loss goals and help you determine the best course of action.  It is also important to note that weight loss surgery is just one tool to help with weight loss and maintenance, and a healthy lifestyle including a balanced diet and regular physical activity is essential for long-term success. | Amputees may be eligible for weight loss surgery, also known as bariatric surgery, depending on their overall health and specific circumstances. Bariatric surgery is typically recommended for people who are severely obese, meaning they have a body mass index (BMI) of 40 or higher or a BMI of 35 or higher with obesity-related health conditions such as type 2 diabetes or sleep apnea. Some amputees may meet these criteria and be candidates for weight loss surgery.  However, there are some factors that may disqualify an amputee from having weight loss surgery. For example, if an amputee has certain medical conditions that would make the surgery too risky, they may not be eligible. Similarly, if an amputee does not have adequate support from friends and family, or if they do not have the ability to make the necessary lifestyle changes after the surgery, they may not be considered a good candidate for the procedure.  It is important for amputees to discuss their weight loss surgery options with their healthcare provider and consider all of the potential risks and benefits before making a decision. | 2, 1 | 1 | 1, 3 |
| Are patients with narcotic addictions eligible for weight loss surgery? | The eligibility for weight loss surgery, including whether a person with a narcotic addiction is a candidate, is determined on a case-by-case basis. Weight loss surgery is generally considered for people who are morbidly obese and have failed to achieve and maintain a healthy weight through non-surgical means, such as diet and exercise. Narcotic addiction may be a factor that is taken into consideration when evaluating a person's suitability for weight loss surgery.  The decision to undergo weight loss surgery is a complex one that involves careful consideration of the potential risks and benefits. It is important for people with narcotic addictions to be honest with their healthcare provider about their substance use, as this may affect the safety and success of the surgery. People with active substance use disorders may not be considered candidates for weight loss surgery until they have received treatment for their addiction and are in recovery.  If you are considering weight loss surgery and have a history of narcotic addiction, it is important to speak with a healthcare provider for a thorough evaluation and guidance on the best course of treatment for you. They can help you understand your options and support you in making an informed decision. | Similar | 1 | 1 | N/A |
| What BMI makes you eligible for bariatric surgery? | Bariatric surgery, which is a type of weight loss surgery, is generally recommended for people who are morbidly obese, meaning that they have a body mass index (BMI) of 40 or higher. People with a BMI of 35 or higher and one or more obesity-related health conditions, such as type 2 diabetes, high blood pressure, or sleep apnea, may also be candidates for bariatric surgery.  BMI is a measure of body fat based on height and weight. It is calculated by dividing a person's weight in kilograms by their height in meters squared. A BMI of 30 or higher is considered obese, while a BMI of 40 or higher is considered morbidly obese.  While BMI is a useful tool for assessing obesity and determining eligibility for bariatric surgery, it is important to note that it is only one factor that is considered. Other factors, such as a person's overall health, medical history, and commitment to lifestyle changes, are also taken into account when deciding if bariatric surgery is appropriate. If you are considering weight loss surgery, it is important to speak with a healthcare provider for a thorough evaluation and guidance on the best course of treatment for you. | Similar | 1 | 1 | NA |
| Is weight loss surgery a ‘cop-out’? To lose weight, should people with obesity just go on a diet and exercise? | Deciding to have weight loss surgery is a personal decision that requires careful consideration of the potential risks and benefits. Weight loss surgery can be an effective tool for helping people with obesity lose weight and improve their overall health, but it is not a "quick fix" or a "cop-out." It is typically recommended for people with a body mass index (BMI) of 40 or higher, or for those with a BMI of 35 or higher who have other health conditions such as type 2 diabetes or sleep apnea. Weight loss surgery can help people with obesity lose weight by limiting the amount of food they can eat or by reducing their absorption of nutrients, but it is not a substitute for healthy lifestyle changes such as eating a healthy diet and engaging in regular physical activity. These behaviors are important for maintaining weight loss and optimizing overall health. | Weight loss surgery is a serious and often life-changing treatment for obesity and related conditions. It is not a "cop-out" or an easy way to lose weight, and it should not be considered lightly. Weight loss surgery is typically only recommended for people who have tried and been unable to lose weight through diet and exercise alone, or for those who have a body mass index (BMI) of 40 or higher, or a BMI of 35 or higher with certain obesity-related health conditions, such as type 2 diabetes or sleep apnea.  That being said, diet and exercise are important components of a healthy weight loss program, and they should be tried before considering weight loss surgery. Losing weight through diet and exercise can improve overall health, reduce the risk of obesity-related medical conditions, and increase the likelihood of long-term success. However, for some people, especially those with severe obesity or certain medical conditions, diet and exercise alone may not be enough to achieve significant and sustained weight loss. In these cases, weight loss surgery may be a safe and effective treatment option.  It is important to remember that weight loss surgery is a tool, not a solution in and of itself. Success after weight loss surgery requires a commitment to making long-term lifestyle changes, including following a healthy diet and getting regular physical activity. It is also important to follow up with your healthcare team and attend support groups to help you stay on track and make the most of your weight loss surgery. | 2, 1 | 2, 1 | N/A |
| Do most people who have weight loss surgery regain their weight? | Weight loss surgery can be an effective tool for helping people with obesity lose weight and improve their overall health. However, like any surgery, weight loss surgery carries certain risks and it is not a guarantee that you will be able to maintain your weight loss long term. Some people may regain a small amount of weight after weight loss surgery, while others may regain more.  There are several factors that can influence the success of weight loss surgery and the ability to maintain weight loss, including:  The type of surgery you had Your adherence to a healthy diet and exercise program Your ability to manage stress and maintain healthy behaviors The presence of any underlying medical conditions Your age and genetics It is important to be realistic about the expectations of weight loss surgery and to understand that it requires a long-term commitment to healthy lifestyle behaviors in order to be successful. Working with your healthcare team and seeking support from friends and family can help you stay on track and maintain your weight loss over time. | Similar | 1 | 1 | N/A |
| Can some medications be stopped after weight loss surgery? | It is possible that you may be able to stop taking certain medications after weight loss surgery, but this will depend on your individual circumstances and the specific medications you are taking. Weight loss surgery can have a significant impact on your weight and overall health, and it is possible that you may no longer need certain medications that you were taking for conditions that are related to obesity. However, it is important to discuss any changes to your medications with your healthcare provider. They will be able to advise you on whether it is safe to stop taking a particular medication and, if so, how to do so safely. Do not stop taking any medications without the guidance of your healthcare provider. It is also important to remember that weight loss surgery is not a "cure-all" and you may still need to take certain medications even after surgery to manage any underlying medical conditions. | It is possible that some medications can be stopped or their dosages adjusted after weight loss surgery, depending on the individual's specific health needs and the medications they are taking. However, it is important to discuss any changes to your medication regimen with your healthcare team before making any changes.  Weight loss surgery can lead to significant improvements in health and may even resolve certain medical conditions, such as type 2 diabetes, high blood pressure, and sleep apnea. As a result, it is possible that some people may no longer need certain medications after surgery. However, each person is different, and the specific medications that can be stopped or adjusted after weight loss surgery will depend on the individual's specific health needs and the medications they are taking.  It is important to remember that medications can play a vital role in managing certain medical conditions and improving overall health. It is important to take your medications as prescribed and not stop or adjust your dosage without consulting with your healthcare team. They can help you understand the potential risks and benefits of adjusting your medication regimen and provide guidance on the best course of action for your individual needs and goals. | 2, 1 | 1 | 1 |
| Is obesity caused by food addiction, similar to alcoholism or drug dependency? | Obesity is a complex condition that can be caused by a variety of factors, including genetics, lifestyle, and environmental factors. While some people may have a genetic predisposition to obesity, this does not mean that they are unable to lose weight or control their weight. Research suggests that food addiction, defined as an inability to control food intake despite negative consequences, may be a contributing factor in some cases of obesity. However, the relationship between food addiction and obesity is not fully understood and is an area of ongoing research.  It is important to note that not everyone who is obese is "addicted" to food, and that obesity is not the same as alcohol or drug dependency. Treatments for obesity, such as weight loss surgery and lifestyle changes, may be different from those used to treat substance abuse disorders. If you are struggling with weight management and are concerned about food addiction, it is important to talk to your healthcare provider for guidance and support. They can help you identify any underlying issues and recommend appropriate treatment options. | Similar | 1 | 1 | N/A |
| Why is weight-loss surgery not a good choice for everyone who is overweight or obese? | Weight loss surgery is not a good choice for everyone who is overweight or obese. Weight loss surgery is typically recommended for people with a body mass index (BMI) of 40 or higher, or for those with a BMI of 35 or higher who have other health conditions such as type 2 diabetes or sleep apnea. It is generally not recommended for people with a BMI of less than 35, unless they have other significant health issues that may be improved by weight loss surgery.  Weight loss surgery is a serious and irreversible procedure that carries certain risks and requires a lifelong commitment to healthy lifestyle behaviors in order to be successful. It is important to carefully consider the potential risks and benefits of weight loss surgery and to discuss your options with your healthcare provider before making a decision. They can help you determine the best approach for managing your weight and optimizing your overall health.  If you are not a candidate for weight loss surgery or if you are not interested in having the procedure, there are other options for managing your weight and improving your overall health. These may include lifestyle changes such as eating a healthy diet, engaging in regular physical activity, and managing stress. Your healthcare provider can provide guidance and support to help you make these changes and achieve your weight loss goals. | Similar | 1 | 1 | N/A |
| What are the different types of weight-loss surgery? | There are several different types of weight loss surgery, also known as bariatric surgery, that can be used to help people with obesity lose weight. The most common types of weight loss surgery include:  Gastric bypass surgery: This procedure involves creating a small pouch at the top of the stomach and attaching it directly to the small intestine. This reduces the amount of food the stomach can hold and limits the absorption of nutrients, leading to weight loss.  Sleeve gastrectomy: This procedure involves removing a large portion of the stomach, leaving a small, tube-shaped stomach. This also reduces the amount of food the stomach can hold and limits the absorption of nutrients, leading to weight loss.  Adjustable gastric band: This procedure involves placing an inflatable band around the upper part of the stomach to create a small pouch. The band can be tightened or loosened to adjust the size of the pouch, which controls the amount of food that can be eaten and helps with weight loss.  Biliopancreatic diversion with duodenal switch: This procedure involves removing a large portion of the stomach and attaching the remaining part of the small intestine directly to the remaining part of the stomach. This reduces the amount of food the stomach can hold and limits the absorption of nutrients, leading to weight loss.  It is important to discuss the different types of weight loss surgery with your healthcare provider to determine which procedure is appropriate for you and to understand the potential risks and benefits of each. | Similar | 1 | 1 | N/A |
| What is the best weight loss surgery to help lose weight and keep it off? | There is no "best" weight loss surgery for everyone, as the optimal procedure for one person may not be suitable for another. The best weight loss surgery for you will depend on your individual circumstances, including your medical history, your weight loss goals, and your overall health.  It is important to discuss the different types of weight loss surgery with your healthcare provider to determine which procedure is appropriate for you and to understand the potential risks and benefits of each. Your healthcare provider will consider factors such as your BMI, your medical history, and your ability to adhere to a healthy diet and exercise program when making a recommendation.  It is also important to keep in mind that weight loss surgery is only one part of the equation when it comes to losing weight and keeping it off. It is important to commit to a healthy lifestyle, including eating a healthy diet, engaging in regular physical activity, and managing stress, in order to maximize the benefits of weight loss surgery and maintain your weight loss over time. Working with your healthcare team and seeking support from friends and family can also help you stay on track and achieve your weight loss goals. | There is no one "best" weight loss surgery that is right for everyone. The decision to have weight loss surgery should be based on a number of factors, including the individual's health, weight, and personal preferences.  Several types of weight loss surgery are available, and each one has its own benefits and risks. Some common types of weight loss surgery include gastric bypass, sleeve gastrectomy, gastric band, and duodenal switch.  Gastric bypass and sleeve gastrectomy are the most common types of weight loss surgery and are generally considered to be very effective at helping people lose a significant amount of weight. These procedures can help to reduce the size of the stomach, which can help to reduce appetite and the amount of food that can be eaten at one time.  Gastric band surgery involves placing an inflatable band around the upper part of the stomach to create a small pouch. The band can be adjusted to control the size of the pouch and the rate at which food passes through the stomach.  Duodenal switch involves removing a large portion of the stomach and reattaching the small intestine to the remaining part of the stomach. This procedure can help to reduce the absorption of calories and can lead to significant weight loss.  It's important to note that weight loss surgery is not a "quick fix" and requires a long-term commitment to lifestyle changes, such as following a healthy diet and getting regular exercise. These changes can help to ensure the best possible outcome and long-term success after weight loss surgery. | 2, 1 | 1 | 2, 1 |
| Will autoimmune disorders affect weight loss after weight loss surgery? | Autoimmune disorders, such as Crohn's disease, ulcerative colitis, and rheumatoid arthritis, can affect the body's ability to absorb nutrients and maintain a healthy weight. Weight loss surgery may be an option for individuals with autoimmune disorders who are struggling to achieve and maintain a healthy weight through diet and exercise alone.  However, it is important to carefully consider the potential risks and benefits of weight loss surgery in the context of an autoimmune disorder. Some autoimmune disorders may make the body more susceptible to certain complications of weight loss surgery, such as malnutrition or infections. It is also possible that weight loss surgery may affect the course or management of the autoimmune disorder.  It is important to discuss your plans for weight loss surgery with your healthcare team and to carefully consider the potential impacts on your autoimmune disorder. Your healthcare team can help you make an informed decision about whether weight loss surgery is right for you and can provide you with guidance on how to manage your condition before and after surgery. | Similar | 1 | 1 | N/A |
| Is it normal to gain some weight back years after weight loss surgery? | It is not uncommon for patients to experience some weight regain after weight loss surgery, particularly in the first few years after the procedure. This can be due to a variety of factors, such as changes in diet and exercise habits, hormonal changes, or other factors that can affect weight management.  Weight regain after weight loss surgery is generally not considered to be a cause for concern unless it is significant or if it is accompanied by other concerning symptoms or behaviors. It is important to communicate with your healthcare team about any changes in your weight or any concerns you may have about your weight management after surgery.  It is generally recommended to follow the guidelines provided by your healthcare team for diet and lifestyle after weight loss surgery to help maintain your weight loss and promote a healthy recovery. This may include eating a balanced diet that is rich in protein, fiber, and nutrients, getting regular physical activity, and managing stress. Your healthcare team can provide you with more specific recommendations based on your individual needs and the type of weight loss surgery you have undergone. | Similar | 1 | 1 | N/A |
| Does a revision from the sleeve to gastric bypass cause more weight loss than the sleeve? | It is generally expected that a revision from the sleeve to gastric bypass will result in greater weight loss than the sleeve surgery alone. This is because the gastric bypass procedure is more restrictive and malabsorptive than the sleeve, which means it can limit the amount of food that can be eaten and absorbed by the body more effectively.  However, the amount of weight loss that can be achieved with a revision from the sleeve to gastric bypass will vary depending on a number of factors, including the individual patient's starting weight, their diet and exercise habits, and their overall health and medical history. It is important to carefully consider the potential risks and benefits of a revision surgery with your healthcare team and to discuss your specific goals and expectations before making a decision about whether to undergo the procedure.  It is generally recommended to follow the guidelines provided by your healthcare team for diet and lifestyle after weight loss surgery to help maintain your weight loss and promote a healthy recovery. This may include eating a balanced diet that is rich in protein, fiber, and nutrients, getting regular physical activity, and managing stress. Your healthcare team can provide you with more specific recommendations based on your individual needs and the type of weight loss surgery you have undergone. | It is possible that a revision from the sleeve to gastric bypass may result in greater weight loss than the sleeve surgery alone. Gastric bypass surgery is generally more effective at promoting weight loss than sleeve surgery, as it involves both restricting the amount of food that can be eaten and disrupting the normal digestive process. However, the amount of weight loss that an individual experiences after a revision from the sleeve to gastric bypass will depend on a variety of factors, including the individual's starting weight, their commitment to making healthy lifestyle changes, and any underlying medical conditions. It is important to discuss the potential benefits and risks of revision surgery with your healthcare team and to carefully consider whether it is the right choice for you. | 1 | 2 | 2, 2 |
| Do patients with chronic acid reflux get a revision from sleeve gastrectomy to gastric bypass? | It is not uncommon for patients who have undergone sleeve gastrectomy to experience persistent or recurrent acid reflux after the surgery. In some cases, a revision from the sleeve to gastric bypass surgery may be recommended to help manage acid reflux and other digestive symptoms.  The decision to undergo a revision from the sleeve to gastric bypass surgery will depend on a number of factors, including the individual patient's health and medical history, their symptoms, and their overall goals for weight loss and management of digestive symptoms. It is important to carefully consider the potential risks and benefits of a revision surgery with your healthcare team and to discuss your specific goals and expectations before making a decision about whether to undergo the procedure.  It is generally recommended to follow the guidelines provided by your healthcare team for diet and lifestyle after weight loss surgery to help manage acid reflux and promote a healthy recovery. This may include avoiding or limiting certain foods or beverages that can exacerbate acid reflux, taking medications as prescribed, and getting regular physical activity. Your healthcare team can provide you with more specific recommendations based on your individual needs and the type of weight loss surgery you have undergone. | Similar | 1 | 1 | N/A |
| Can you have weight loss surgery if you're on medication that can cause weight gain? | In some cases, patients who are taking medications that can cause weight gain may be eligible for weight loss surgery, depending on the specific medication and the individual patient's health and medical history.  It is important to carefully consider the potential risks and benefits of weight loss surgery with your healthcare team and to discuss any medications you are taking that may affect your weight. Your healthcare team can provide you with guidance on whether weight loss surgery is appropriate for you and can help you to explore options for managing your weight while on medication.  It is generally recommended to follow the guidelines provided by your healthcare team for diet and lifestyle after weight loss surgery to help maintain your weight loss and promote a healthy recovery. This may include eating a balanced diet that is rich in protein, fiber, and nutrients, getting regular physical activity, and managing stress. Your healthcare team can provide you with more specific recommendations based on your individual needs and the type of weight loss surgery you have undergone. | Similar | 1 | 1 | N/A |
| Will weight loss surgery lower blood pressure? | Weight loss surgery, such as gastric bypass or sleeve gastrectomy, can lead to significant weight loss, which can in turn lower blood pressure. High blood pressure, or hypertension, is a common condition that increases the risk of heart disease, stroke, and other serious health problems. Losing weight can help lower blood pressure and improve overall health.  It is important to note that weight loss surgery is not a substitute for lifestyle changes, such as following a healthy diet and getting regular exercise, which are also important for maintaining a healthy blood pressure.  If you have high blood pressure and are considering weight loss surgery, it is important to talk to your healthcare provider. They can help you understand the potential risks and benefits of the surgery and recommend the best course of treatment for you. | Weight loss surgery can lead to significant weight loss and can help improve many health conditions, including high blood pressure (hypertension). In fact, research has shown that weight loss surgery can be an effective treatment for hypertension in people who are obese or have morbid obesity.  After weight loss surgery, it is common for people to experience a significant decrease in blood pressure, as well as improvements in other cardiovascular risk factors such as cholesterol levels and blood sugar control. These improvements can help reduce the risk of developing serious health problems such as heart disease, stroke, and diabetes.  If you have hypertension and are considering weight loss surgery, it is important to discuss your blood pressure and other health conditions with your surgeon or healthcare provider. They can help determine the best course of action for you and can provide guidance on how to manage your blood pressure after surgery. | 2, 1 | 1 | 1 |
| Which surgery will cause the greatest weight loss: sleeve gastrectomy or gastric bypass? | Both sleeve gastrectomy and gastric bypass are effective weight loss surgeries that can lead to significant weight loss. However, the amount of weight loss that can be achieved with either surgery can vary widely depending on a number of factors, including the individual's starting weight, age, and overall health.  On average, people who have gastric bypass surgery may lose more weight than those who have sleeve gastrectomy. Gastric bypass surgery works by reducing the size of the stomach and rerouting the small intestine to bypass a portion of the stomach, which can lead to more significant weight loss.  Sleeve gastrectomy involves removing a portion of the stomach to create a small, tube-like stomach. While weight loss with sleeve gastrectomy may not be as rapid as with gastric bypass, it can still be substantial and can lead to significant improvements in health.  Ultimately, the best weight loss surgery for you will depend on your individual circumstances and the recommendations of your healthcare team. It is important to discuss the potential risks and benefits of each surgery with your healthcare provider to determine the best course of treatment for you. | Similar | 1 | 1 | N/A |
| Is bariatric surgery necessary or can weight be lost with only a restrictive diet? | Bariatric surgery, also known as weight loss surgery, is a type of procedure that is used to help people with obesity or severe obesity lose weight. It is generally recommended for people who have been unable to achieve significant and sustained weight loss through diet and exercise alone.  Weight loss surgery works by limiting the amount of food that the stomach can hold, which can help people lose weight. It can also alter the hormones and brain signals that regulate appetite and hunger, which can make it easier to stick to a healthy diet and maintain weight loss.  While weight loss surgery can be an effective tool for achieving significant and sustained weight loss, it is not right for everyone. It is a major surgery that carries risks and requires a lifelong commitment to healthy lifestyle changes, such as following a healthy diet and getting regular exercise.  For some people, a restrictive diet may be enough to achieve weight loss goals. However, it is important to work with a healthcare provider or registered dietitian to develop a safe and effective weight loss plan that meets your individual needs and goals. They can help you understand the potential risks and benefits of different treatment options and recommend the best course of action for you. | Similar | 1 | 1 | N/A |
| How much weight is normally lost in the first month after a sleeve gastrectomy? | Weight loss after sleeve gastrectomy can vary significantly from person to person. Some people may lose a significant amount of weight in the first month after surgery, while others may lose less. The amount of weight loss that a person can expect after sleeve gastrectomy typically depends on a number of factors, including the person's starting weight, age, gender, and overall health.  On average, people tend to lose about 50-60% of their excess body weight within the first six months to one year after sleeve gastrectomy. This means that if you are 100 pounds (45 kg) overweight, you can expect to lose about 50-60 pounds (22-27 kg) in the first six months to one year after surgery. However, it is important to note that weight loss after sleeve gastrectomy is not a one-size-fits-all process, and the amount of weight that you lose may be different from others.  It is also important to note that weight loss after sleeve gastrectomy is not immediate, and it may take several weeks or even months before you start to see significant changes in your weight. It is important to be patient and to follow the instructions of your surgeon and medical team to ensure a successful recovery and to maximize your weight loss results. | Similar | 1 | 1 | N/A |
| Will bariatric surgery cure diabetes? | Bariatric surgery can be an effective treatment for diabetes, but it is not a cure. Diabetes is a chronic condition that affects the way your body processes sugar (glucose), and it requires ongoing management to keep it under control.  Bariatric surgery, such as gastric bypass or sleeve gastrectomy, can help to improve diabetes by promoting weight loss and improving insulin sensitivity. These procedures can cause significant and rapid weight loss, which can help to improve the body's ability to use insulin and lower blood sugar levels. In many cases, people who have bariatric surgery experience a significant improvement in their diabetes within the first few weeks or months after surgery.  However, it is important to note that bariatric surgery is not a magic cure for diabetes. While it can help to improve diabetes and may even lead to a complete resolution of the condition in some cases, it is not a guarantee. People who have had bariatric surgery will still need to manage their diabetes by following a healthy diet, getting regular exercise, and taking medications as prescribed by their doctor. It is also important to continue monitoring your blood sugar levels and to work with your healthcare team to manage your diabetes over the long term. | Similar | 1 | 1 | N/A |
| Will bariatric surgery lower cholesterol? | Bariatric surgery can help to lower cholesterol levels in some people. Cholesterol is a type of fat (lipid) that is found in the bloodstream and in all of the cells in your body. It is an important component of cell membranes, and it is also used to make hormones, vitamin D, and bile acids that help to digest fats.  However, high levels of cholesterol in the blood can increase the risk of heart disease and stroke. There are two main types of cholesterol: low-density lipoprotein (LDL) cholesterol, often referred to as "bad" cholesterol, and high-density lipoprotein (HDL) cholesterol, often referred to as "good" cholesterol.  Bariatric surgery, such as gastric bypass or sleeve gastrectomy, can help to lower cholesterol levels by promoting weight loss and improving insulin sensitivity. These procedures can cause significant and rapid weight loss, which can help to lower LDL cholesterol levels and increase HDL cholesterol levels. In many cases, people who have bariatric surgery experience a significant improvement in their cholesterol levels within the first few weeks or months after surgery.  However, it is important to note that bariatric surgery is not a guarantee of improved cholesterol levels. The specific effects of the surgery on cholesterol levels can vary from person to person, and it is important to work with your healthcare team to manage your cholesterol levels over the long term. This may involve making lifestyle changes, such as eating a healthy diet and getting regular exercise, and taking medications as prescribed by your doctor. | Similar | 1 | 1 | N/A |
| How is the mini gastric sleeve different than the regular sleeve gastrectomy? | The mini gastric sleeve is a type of weight loss surgery that is similar to the regular sleeve gastrectomy, but it is a shorter and less invasive procedure. Both the mini gastric sleeve and the regular sleeve gastrectomy involve the removal of a portion of the stomach to create a smaller, sleeve-shaped stomach. This smaller stomach can hold less food and may help to reduce the amount of food that a person eats, leading to weight loss.  The main difference between the mini gastric sleeve and the regular sleeve gastrectomy is the extent of the surgery. The mini gastric sleeve is a shorter procedure that involves the removal of a smaller portion of the stomach. As a result, it is typically less invasive and has a shorter recovery time than the regular sleeve gastrectomy.  However, it is important to note that the mini gastric sleeve may not be as effective at promoting weight loss as the regular sleeve gastrectomy. It may also be associated with a higher risk of complications, such as leaks or strictures (narrowing) in the stomach. The specific risks and benefits of the mini gastric sleeve will depend on the individual patient and should be carefully considered before deciding which type of surgery is right for you. If you are considering weight loss surgery, it is important to speak with a medical professional to discuss your options and to determine the best course of treatment for your specific needs and goals. | The mini gastric sleeve, also known as the single anastomosis gastric sleeve or the mini-sleeve, is a type of weight loss surgery that is similar to the regular sleeve gastrectomy. Both procedures involve the removal of a portion of the stomach to create a smaller, sleeve-shaped stomach. However, there are some key differences between the two procedures.  One difference is the size of the stomach that is created. The mini gastric sleeve procedure involves the removal of a smaller portion of the stomach, resulting in a smaller stomach size than is achieved with a regular sleeve gastrectomy. Another difference is the method of attaching the remaining stomach to the small intestine. In the mini gastric sleeve procedure, the remaining stomach is attached to the small intestine using a single anastomosis, or connection, whereas in the regular sleeve gastrectomy, the stomach is attached to the small intestine using two separate anastomoses.  Both the mini gastric sleeve and the regular sleeve gastrectomy are restrictive weight loss procedures that can help individuals to lose weight by reducing the amount of food that can be comfortably eaten at one time. However, the mini gastric sleeve procedure may be associated with a slightly higher risk of complications compared to the regular sleeve gastrectomy, and the long-term effectiveness of the procedure is not well-established. If you are considering weight loss surgery, it is important to discuss the potential risks and benefits of all available procedures with your healthcare provider and to carefully consider which procedure is right for you. | 1, 4 | 2, 4 | 4, 4 |
| What type of bariatric surgery is best for someone with polycystic ovarian syndrome? | The type of bariatric surgery that is best for someone with polycystic ovarian syndrome (PCOS) will depend on a number of factors, including the person's overall health, weight, and specific goals. PCOS is a hormonal disorder that affects the ovaries and can cause irregular periods, excess hair growth, weight gain, and fertility problems. It is often associated with obesity, and losing weight can be an important part of managing the condition.  Bariatric surgery, such as gastric bypass or sleeve gastrectomy, can be an effective treatment for obesity and may help to improve symptoms of PCOS. These procedures can cause significant and rapid weight loss, which can help to improve insulin sensitivity, reduce testosterone levels, and restore regular periods in people with PCOS.  However, it is important to note that bariatric surgery is not a magic cure for PCOS, and it is not appropriate for everyone. The specific risks and benefits of the surgery will depend on the individual patient and should be carefully considered before deciding which type of surgery is right for you. If you are considering bariatric surgery and have PCOS, it is important to speak with a medical professional to discuss your options and to determine the best course of treatment for your specific needs and goals. | There is no one "best" type of bariatric surgery for someone with polycystic ovary syndrome (PCOS). The type of surgery that is most appropriate for you will depend on a number of factors, including your specific health needs, your body mass index (BMI), and your overall health.  Gastric bypass surgery and gastric sleeve surgery are two common types of bariatric surgery that may be considered for people with PCOS. Both of these procedures can help you lose weight and improve the symptoms of PCOS, such as irregular periods, excess hair growth, and fertility problems.  However, it's important to note that bariatric surgery is a major procedure and is not a suitable option for everyone. It's important to discuss all of your treatment options with your doctor and a registered dietitian to determine the best approach for your specific needs. | 1, 2 | 1 | 1 |
| What is the most common weight loss surgery? | The most common weight loss surgeries are gastric bypass, sleeve gastrectomy, and adjustable gastric banding.  Gastric bypass surgery is a procedure in which the stomach is divided into a small upper pouch and a larger lower pouch. The small intestine is then divided and one end is connected to the upper pouch, while the other end is connected to the lower pouch. This creates a bypass around the stomach and the first section of the small intestine, which helps to reduce the amount of food you can eat and the number of calories and nutrients your body absorbs.  Sleeve gastrectomy is a procedure in which a large portion of the stomach is removed, leaving a small, sleeve-shaped stomach. This surgery helps to reduce the amount of food you can eat and can also alter hormones and brain signals that control hunger and fullness.  Adjustable gastric banding is a procedure in which an inflatable silicone band is placed around the upper part of the stomach. The band can be tightened or loosened to adjust the size of the stomach and help control the amount of food you can eat.  It is important to discuss the potential risks and benefits of each type of weight loss surgery with a healthcare professional to determine which procedure may be the best option for you based on your individual situation and needs. | The most common types of weight loss surgery, also known as bariatric surgery, are:  Gastric bypass: This procedure involves creating a small pouch in the stomach and bypassing a portion of the small intestine. This helps to reduce the amount of food that can be eaten and the amount of calories and nutrients that are absorbed.  Gastric sleeve: This procedure involves removing a large portion of the stomach, leaving a small, sleeve-shaped stomach. This helps to reduce the amount of food that can be eaten and the production of hunger-inducing hormones.  Adjustable gastric band: This procedure involves placing an adjustable band around the upper portion of the stomach to create a small pouch. The size of the pouch can be adjusted by inflating or deflating the band.  It's worth noting that there are pros and cons to each type of weight loss surgery, and the best option for you will depend on your individual needs and circumstances. It's important to discuss all of your options with a healthcare professional to determine the best approach for your specific needs. | 3, 1 | 1 | 3, 1 |
| Will weight loss surgery cure sleep apnea? | Weight loss surgery may help to improve or resolve sleep apnea in some individuals. Sleep apnea is a condition in which a person's breathing is repeatedly interrupted during sleep, leading to poor sleep quality and an increased risk of medical conditions such as high blood pressure, heart disease, and diabetes.  Obesity is a major risk factor for sleep apnea, and weight loss surgery can help to promote significant weight loss, which can in turn help to improve or resolve sleep apnea in some individuals. In fact, research has shown that weight loss surgery can be an effective treatment for sleep apnea, especially in individuals who are severely obese.  It is important to note, however, that weight loss surgery is not a guaranteed cure for sleep apnea, and some individuals may still require additional treatment, such as the use of a continuous positive airway pressure (CPAP) machine, to manage the condition. It is a good idea to discuss your specific situation with a healthcare professional to determine the best course of treatment for your sleep apnea. | Similar | 1 | 1 | N/A |
| What are realistic weight loss goals after bariatric surgery? | Realistic weight loss goals after bariatric surgery will vary depending on your starting weight, the type of surgery you had, and your individual circumstances.  In general, it is common for people to lose a significant amount of weight after bariatric surgery, with many people losing 50-75% of their excess body weight within the first year after surgery. However, weight loss may vary from person to person and may be slower in the long term.  It is important to remember that weight loss surgery is just one tool to help with weight loss and maintenance, and a healthy lifestyle including a balanced diet and regular physical activity is essential for long-term success. It is also important to work with a healthcare professional to develop realistic weight loss goals based on your individual needs and circumstances.  It is also a good idea to focus on non-scale victories, such as improvements in health markers, increased energy levels, and improved self-esteem, rather than just the number on the scale. These can be important indicators of progress and can help you stay motivated on your weight loss journey. | Similar | 1 | 1 | N/A |
| Will weight loss surgery cure insulin resistance? | Weight loss surgery may help to improve or resolve insulin resistance in some individuals. Insulin resistance is a condition in which the body's cells do not respond properly to insulin, leading to high blood sugar levels and an increased risk of type 2 diabetes. Obesity is a major risk factor for insulin resistance, and weight loss surgery can help to promote significant weight loss, which can in turn help to improve insulin resistance in some individuals.  In fact, research has shown that weight loss surgery can be an effective treatment for insulin resistance and type 2 diabetes, especially in individuals who are severely obese. However, it is important to note that weight loss surgery is not a guaranteed cure for insulin resistance, and some individuals may still require additional treatment, such as medication, to manage the condition.  It is also important to remember that weight loss surgery is just one tool to help with weight loss and maintenance, and a healthy lifestyle including a balanced diet and regular physical activity is essential for long-term success. | Similar | 1 | 1 | N/A |
| Does weight loss surgery prolong lifespan? | There is evidence to suggest that weight loss surgery can improve lifespan in people who are obese. Obesity is associated with a number of serious health conditions, including heart disease, diabetes, and certain types of cancer, which can significantly shorten lifespan. By helping people lose weight and reduce their risk of these conditions, weight loss surgery may be able to improve their chances of living a longer, healthier life.  However, it is important to note that weight loss surgery is not a magic bullet and does not guarantee a longer lifespan. The best way to improve your chances of living a long and healthy life is to adopt a healthy lifestyle, which includes a balanced diet and regular physical activity. Weight loss surgery can be an effective tool to help people achieve and maintain a healthy weight, but it is only one part of the equation. | There is evidence to suggest that weight loss surgery can improve lifespan, particularly in individuals who are severely obese. Severe obesity is defined as having a body mass index (BMI) of 40 or higher, or having a BMI of 35 or higher and experiencing obesity-related health problems such as diabetes, high blood pressure, or sleep apnea. Studies have shown that weight loss surgery can significantly reduce the risk of premature death in people with severe obesity.  For example, a study published in the New England Journal of Medicine found that bariatric surgery was associated with a 40% reduction in all-cause mortality among obese individuals with a BMI of 35 or higher. Another study published in the journal JAMA found that bariatric surgery was associated with a 29% reduction in all-cause mortality among obese individuals with a BMI of 40 or higher.  It is important to note that weight loss surgery is not a magic cure, and it is not appropriate for everyone. It is a major surgical procedure that carries risks and requires a significant commitment to lifestyle changes in order to be successful. It is important to discuss the potential benefits and risks of weight loss surgery with a qualified healthcare provider before making a decision. | 2, 1 | 1 | 2, 1 |
| Will weight loss surgery improve urinary incontinence? | Weight loss surgery may improve urinary incontinence in some people who are obese. Obesity can cause or exacerbate urinary incontinence in a number of ways. For example, excess weight can put pressure on the bladder and surrounding muscles, making it more difficult to control urination. Obesity can also lead to hormonal changes that can affect bladder function.  By helping people lose weight, weight loss surgery may be able to reduce the pressure on the bladder and improve the function of the muscles and hormones involved in urinary control. However, the extent to which weight loss surgery can improve urinary incontinence will vary depending on the individual and the underlying causes of their incontinence. In some cases, additional treatment or lifestyle modifications may be needed to fully address the problem. If you are experiencing urinary incontinence and are considering weight loss surgery, it is important to discuss your concerns with a healthcare provider. | Similar | 1 | 1 | N/A |
| **Preoperative Preparation (2)** | | | | | |
| Will you have to go on a diet before I have weight loss surgery? | It is generally recommended that people considering weight loss surgery try to lose weight through diet and exercise before undergoing the procedure. This can help reduce the risk of complications during surgery and improve the chances of success. Your healthcare team may recommend that you follow a specific diet or nutrition plan before surgery to help you lose weight and prepare for the procedure. This may involve making healthier food choices, such as eating more fruits and vegetables, lean proteins, and whole grains, and cutting back on processed and sugary foods. It may also involve monitoring your portion sizes and tracking your food intake to ensure you are getting the nutrients you need while still losing weight. Your healthcare team can provide guidance and support to help you make these lifestyle changes and prepare for surgery. | Similar | 2 | 1 | 1 |
| What can you do before weight loss surgery to lower the risk of complications? | There are several things you can do before weight loss surgery to lower the risk of complications. These may include:  Quitting smoking: Smoking can increase the risk of complications after surgery, including infections, wound healing problems, and lung problems. If you smoke, it is important to quit at least four to six weeks before surgery to reduce your risk of complications.  Losing weight: Losing weight before surgery can help reduce the size of your liver and make the surgery safer. Your healthcare provider may recommend a weight loss plan to help you lose weight before surgery.  Managing chronic conditions: If you have chronic health conditions such as diabetes or high blood pressure, it is important to work with your healthcare team to optimize your health before surgery. This may involve adjusting your medications, following a special diet, or making other lifestyle changes.  Reducing stress: Stress can have negative effects on your health and can increase the risk of complications after surgery. It is important to find ways to manage stress before surgery, such as through relaxation techniques, exercise, or support from friends and family.  By taking these steps before surgery, you can help lower your risk of complications and optimize your chances of a successful outcome. It is also important to follow your healthcare team's instructions and to discuss any concerns you have with them. They can provide guidance and support to help you prepare for surgery and achieve your weight loss goals. | There are several things that you can do before weight loss surgery to lower the risk of complications. Here are some tips:  Get a medical evaluation: It's important to have a thorough medical evaluation before weight loss surgery to identify any potential issues that could increase the risk of complications.  Quit smoking: If you smoke, it's important to quit before weight loss surgery. Smoking can increase the risk of complications such as infection and poor healing.  Get vaccinated: Some vaccinations, such as the flu shot and the pneumonia vaccine, can help to reduce the risk of complications after weight loss surgery.  Follow a healthy diet: Eating a healthy diet before weight loss surgery can help to improve overall health and may reduce the risk of complications.  Attend a pre-surgery education class: Many weight loss surgery programs offer pre-surgery education classes that can help to prepare you for the procedure and recovery.  Follow your surgeon's instructions: It's important to follow your surgeon's instructions before weight loss surgery, such as any special instructions for eating or drinking, to help reduce the risk of complications.  By taking these steps, you can help to ensure the best possible outcome and a smooth recovery after weight loss surgery. | 3, 1 | 2 | 3, 3 |
| For which medical problems (such as diabetes, heart disease, or high blood pressure) do you need to see the doctor before the weight loss surgery? | If you have certain medical conditions, it is important to see your healthcare provider before undergoing weight loss surgery. These conditions may include:  Diabetes: If you have type 2 diabetes, it is important to work with your healthcare team to optimize your blood sugar control before surgery. Poorly controlled diabetes can increase the risk of complications during and after surgery.  Heart disease: If you have heart disease, it is important to see your healthcare provider before surgery to assess your heart health and determine if you are a good candidate for surgery.  High blood pressure: If you have high blood pressure, it is important to work with your healthcare team to optimize your blood pressure control before surgery. High blood pressure can increase the risk of complications during and after surgery.  Sleep apnea: If you have sleep apnea, it is important to see your healthcare provider before surgery to assess your condition and determine if you are a good candidate for surgery.  It is important to discuss any medical conditions you have with your healthcare provider before weight loss surgery to determine if you are a good candidate for the procedure and to identify any potential risks or concerns. They can provide guidance and support to help you prepare for surgery and optimize your chances of a successful outcome. | Similar | 1 | 1 | N/A |
| What medicines should you take on the day of weight loss surgery? Are there any medicines that you should not take? | On the day of weight loss surgery, it is important to follow your healthcare team's instructions for taking medications. In general, you should take any medications that your healthcare provider has prescribed for you, unless you are instructed otherwise. You should also inform your healthcare team about any over-the-counter medications, supplements, or herbs that you are taking, as these may need to be stopped or adjusted before surgery.  It is important to avoid taking any medications or supplements that may increase the risk of bleeding during surgery, such as aspirin, non-steroidal anti-inflammatory drugs (NSAIDs), or vitamin E. Your healthcare provider will provide specific instructions about which medications to take or avoid on the day of surgery.  It is also important to inform your healthcare team about any allergies you have or any medications that you are unable to take. This will help them determine which medications are safe for you to take before and after surgery.  It is important to follow your healthcare team's instructions for taking medications on the day of surgery to ensure a safe and successful procedure. If you have any questions or concerns about your medications, it is important to discuss them with your healthcare provider. | Similar | 1 | 1 | N/A |
| Do you need to see different specialists other than the surgeon before weight loss surgery? | Yes, it is common for patients to see a team of healthcare providers before undergoing weight loss surgery. This may include a primary care physician, a surgeon who specializes in weight loss surgery, a nutritionist, a mental health provider, and possibly other specialists depending on the patient's individual needs and medical history.  It is important to have a thorough evaluation before weight loss surgery to ensure that the patient is a good candidate for the procedure and to identify any medical conditions that need to be addressed before or after surgery. The evaluation may include a physical examination, laboratory tests, and imaging studies, as well as a review of the patient's medical history and lifestyle. The healthcare team will also discuss the potential risks and benefits of the procedure with the patient and help the patient make an informed decision about whether weight loss surgery is right for them. | Similar | 1 | 1 | N/A |
| Will having suicidal thoughts stop you from being scheduled for weight loss surgery even if you are under the care of a psychiatrist and go to counseling regularly? | Suicidal thoughts are a serious concern, and it is important to seek help and support if you are experiencing these thoughts. It is not uncommon for individuals with obesity to experience mental health issues such as depression, anxiety, and body image concerns, and these issues may need to be addressed before or after weight loss surgery.  If you are experiencing suicidal thoughts and are seeking care from a mental health provider, it is important to discuss your concerns with your healthcare team. They may recommend that you delay weight loss surgery until your mental health has improved or that you continue to receive treatment before and after surgery to help manage your symptoms.  The decision to undergo weight loss surgery is a complex one, and it is important to carefully consider the potential risks and benefits of the procedure. Your healthcare team can help you make an informed decision about whether weight loss surgery is right for you, and they can provide you with support and guidance throughout the process.  If you are experiencing suicidal thoughts or if you are in crisis, it is important to seek immediate help. You can call the National Suicide Prevention Lifeline at 1-800-273-TALK (8255) or text HOME to the Crisis Text Line at 741741 to speak with a trained crisis counselor. These services are available 24 hours a day, 7 days a week, and are free and confidential. | It is important to address any mental health issues, including suicidal thoughts, before undergoing any type of surgery. If you are currently experiencing suicidal thoughts and are under the care of a mental health professional, it is important to discuss this with your surgeon and follow the recommendations of your mental health provider. They may recommend that you postpone surgery until your mental health has improved or that you continue with your mental health treatment in conjunction with surgery. It is important to prioritize your mental and physical health and to make sure that you are in a good place before undergoing any type of surgery. | 1, 2 | 1 | 1, 2 |
| Are there any foods to avoid one week before weight loss surgery? | It is generally recommended to avoid certain foods in the week leading up to weight loss surgery to help reduce the risk of complications during the procedure. Your healthcare team will provide you with specific instructions on what to eat and drink before surgery, as the guidelines may vary depending on the type of surgery you are undergoing and your individual needs.  In general, it is recommended to avoid foods that are high in fat or sugar in the week before weight loss surgery, as these can increase the risk of complications such as nausea, vomiting, and diarrhea. It is also a good idea to avoid foods that are hard to digest, as they may cause discomfort or bloating.  Some specific examples of foods to avoid in the week before weight loss surgery may include:  Fried foods High-fat meats, such as bacon, sausage, and ribs Full-fat dairy products, such as cheese and whole milk Pastries and baked goods Sugary drinks, such as soda and fruit juices It is important to follow the guidelines provided by your healthcare team carefully and to avoid eating and drinking anything that is not recommended in the week before surgery. If you have any questions or concerns, it is important to contact your healthcare team for guidance. | Similar | 1 | 1 | N/A |
| Is a liquid diet required before weight loss surgery? | It is generally recommended to follow a liquid diet in the days or weeks leading up to weight loss surgery to help reduce the risk of complications during the procedure. A liquid diet may help to reduce the amount of food in the gastrointestinal tract, which can make the surgery easier to perform and help reduce the risk of complications.  The specific guidelines for a liquid diet before weight loss surgery may vary depending on the type of surgery you are undergoing and your individual needs. In general, a liquid diet may include:  Water Broth Sugar-free drinks, such as coffee, tea, and clear soda Low-fat, low-sugar protein shakes or supplements It is important to follow the guidelines provided by your healthcare team carefully and to avoid eating or drinking anything that is not recommended in the days or weeks before surgery. If you have any questions or concerns, it is important to contact your healthcare team for guidance.  It is also important to stay well-hydrated during this time, as a liquid diet may not provide all the fluids and nutrients that your body needs. Your healthcare team can provide you with guidance on how to stay hydrated and nourished while following a liquid diet. | Similar | 1 | 1 | N/A |
| How important is it to have family support before undergoing bariatric surgery? | Having supportive family and friends can be beneficial for anyone undergoing weight loss surgery, as it can provide a sense of emotional and practical support during the pre-surgery process and throughout the recovery period.  However, it is important to remember that ultimately, the decision to undergo weight loss surgery is a personal one and should be based on your own goals, values, and needs. It is important to carefully consider the potential risks and benefits of the procedure and to make an informed decision that is right for you.  If you do decide to undergo weight loss surgery, it is generally recommended to involve your family and friends in the process as much as possible. This can help to ensure that you have a strong support system in place to help you manage the physical and emotional challenges that may arise before and after the procedure.  It is also important to communicate with your healthcare team about any concerns or questions you may have about weight loss surgery and to seek support from them as needed. They can provide you with guidance and support throughout the process and can help you to make an informed decision about whether weight loss surgery is right for you. | Similar | 1 | 1 | N/A |
| Is it normal to get migraines with the pre-op liquid diet before bariatric surgery? | It is not uncommon for patients to experience migraines or other types of headaches while on a pre-op liquid diet before weight loss surgery. This can be due to a variety of factors, including changes in diet, dehydration, or changes in the body's metabolism.  If you are experiencing migraines while on a pre-op liquid diet, it is important to communicate with your healthcare team about your symptoms. They can help to identify the cause of your migraines and provide you with appropriate treatment. Treatment options may include medications, lifestyle changes, or other interventions.  It is generally recommended to follow the guidelines provided by your healthcare team for the pre-op liquid diet and for managing migraines. This may include drinking plenty of fluids to help stay hydrated, getting regular physical activity, and managing stress. Your healthcare team can provide you with more specific recommendations based on your individual needs and the type of weight loss surgery you are preparing for. | Similar | 1 | 1 | N/A |
| Can you use marijuana before weight loss surgery? | Marijuana, also known as cannabis, is a psychoactive drug that is derived from the cannabis plant. It is commonly used for recreational and medicinal purposes, but it can also have potential side effects and risks.  It is generally not recommended to use marijuana before weight loss surgery. In fact, most weight loss surgery programs require patients to stop using marijuana and other substances at least six weeks before the surgery.  This is because marijuana can affect the body's ability to heal and can interfere with the effectiveness of certain medications. It can also cause impairments in judgment, coordination, and other important functions that are necessary for a safe and successful surgery.  If you are considering weight loss surgery and currently use marijuana, it is important to discuss this with your healthcare provider. They can help you understand the potential risks and recommend the best course of action for you. | Similar | 1 | 1 | N/A |
| Why is the liver-shrinking diet necessary before weight loss surgery? | The liver-shrinking diet, also known as the pre-surgery diet, is often recommended before weight loss surgery to help reduce the size of the liver. This is because a larger liver can make weight loss surgery more difficult and increase the risk of complications.  The liver-shrinking diet typically consists of a low-calorie, low-fat diet that is high in protein. It is typically followed for a period of two to three weeks before weight loss surgery. The specific guidelines for the liver-shrinking diet may vary depending on the type of weight loss surgery you are having and your individual needs.  It is important to follow the pre-surgery diet and other instructions provided by your surgeon to help ensure the success and safety of your weight loss surgery. It is also important to note that weight loss surgery is just one tool to help with weight loss and maintenance, and a healthy lifestyle including a balanced diet and regular physical activity is essential for long-term success. | Similar | 1 | 1 | N/A |
| Do IUDs have to be removed before having weight loss surgery? | It is generally not necessary to remove an IUD (intrauterine device) before having weight loss surgery. An IUD is a small, T-shaped device that is inserted into the uterus to prevent pregnancy. It is a long-acting, reversible form of contraception that is effective for several years.  However, it is important to inform your surgeon and other members of your healthcare team if you have an IUD before having weight loss surgery. This is because the IUD may need to be removed or temporarily removed during the surgery, depending on the type of weight loss surgery you are having and the location of the IUD.  It is also a good idea to discuss your contraception options with your healthcare team before and after weight loss surgery to ensure that you are using a method that is safe and appropriate for your specific situation. | It is generally recommended to remove an intrauterine device (IUD) before having weight loss surgery. An IUD is a small, T-shaped device that is inserted into the uterus to prevent pregnancy. There are two types of IUDs: hormonal and copper. Both types are considered safe and effective for most people, but there are some potential risks and considerations to be aware of when having an IUD removed before weight loss surgery.  One potential risk is the possibility of the IUD becoming displaced during the surgery. This can happen if the IUD is not properly positioned or if the uterus is manipulated during the surgery. If the IUD becomes displaced, it may no longer be effective at preventing pregnancy.  Another consideration is the potential for bleeding during or after the surgery. Weight loss surgery, especially laparoscopic procedures, can involve the use of instruments that may cause bleeding or injury to the uterus or cervix. If an IUD is present, there is a risk that it may become dislodged or cause bleeding during the surgery.  For these reasons, it is generally recommended to remove the IUD before having weight loss surgery. If you are planning to have weight loss surgery and have an IUD, it is important to discuss your options with your healthcare provider. They will be able to help you determine the best course of action based on your individual circumstances. | 1, 3 | 1, 3 | N/A |
| What items should be taken to the hospital on the day of weight loss surgery? | It is a good idea to bring the following items with you to the hospital on the day of weight loss surgery:  A list of your current medications, including the dosage and frequency of each medication. A copy of your advance directive or living will, if you have one. A list of any allergies you have. Comfortable clothing and shoes to wear home after surgery. Personal care items, such as a toothbrush, toothpaste, and deodorant. A phone and charger. Any other items that you may need during your hospital stay, such as eyeglasses or hearing aids. It is also a good idea to arrange for someone to drive you home after surgery and to stay with you for the first few days to help with post-surgery care.  It is important to follow the pre-surgery instructions provided by your surgeon and to bring any other items that they may have recommended. It is also a good idea to speak with your surgeon or a healthcare professional if you have any questions or concerns about what to bring to the hospital on the day of your surgery. | It's a good idea to bring the following items with you to the hospital on the day of your weight loss surgery:  Photo identification, such as a driver's license or passport Insurance card List of current medications you are taking, including dosage information Payment for any copays or deductibles Loose, comfortable clothing to wear after the surgery Books, magazines, or other forms of entertainment to pass the time while you are in the hospital A pillow or blanket, if you have a preferred one Toothbrush and other personal hygiene items It's also a good idea to make arrangements for someone to drive you home from the hospital after your surgery. You will not be able to drive yourself home due to the effects of the anaesthesia. | 2, 2 | 1 | 1 |
| When are blood thinners recommended before weight loss surgery? | Blood thinners, also known as anticoagulants, may be recommended before weight loss surgery in certain situations to help reduce the risk of blood clots. Blood clots can occur after surgery, particularly in people who are immobilized or have certain risk factors, such as a history of blood clots, a sedentary lifestyle, or obesity. Blood clots can be serious and even life-threatening if they break off and travel to the lungs (pulmonary embolism).  There are several different types of blood thinners that may be used before surgery, including heparin, low molecular weight heparin, and warfarin. The specific blood thinner and dosing regimen that is recommended will depend on the individual and their specific needs. In some cases, blood thinners may be started a few days before surgery and continued for a period of time after the procedure.  If you are considering weight loss surgery and have a history of blood clots or other risk factors for blood clots, it is important to discuss the use of blood thinners with your healthcare provider. They will be able to determine if blood thinners are appropriate for you and, if so, which type and dosage is most appropriate. | It is common for people to be prescribed blood thinners before certain types of surgery, including weight loss surgery, in order to help prevent blood clots from forming. Blood clots can occur after surgery, particularly in people who are inactive for long periods of time, and they can be dangerous because they can travel to the lungs and cause a condition called pulmonary embolism, which can be life-threatening.  The decision to prescribe blood thinners before weight loss surgery will depend on a number of factors, including the type of surgery being performed and the individual's medical history. Some people who may be at higher risk of developing blood clots after surgery may be more likely to be prescribed blood thinners as a preventive measure.  If you are scheduled to have weight loss surgery and have concerns about blood thinners, it is important to speak with your healthcare provider. They will be able to provide specific recommendations based on your individual circumstances and medical history. | 1 | 1, 3 | 1, 3 |
| **Recovery, Risks, and Complications (3)** | | | | | |
| How much time off of work is recommended after weight loss surgery? | The amount of time off work that is recommended after weight loss surgery may vary depending on the type of surgery you have and your individual circumstances. Some people may be able to return to work within a few days, while others may need several weeks or more to recover. It is important to follow the recommendations of your healthcare team and to take the time you need to rest and recover properly. Factors that may affect the length of time off work may include: - The type of surgery you had (laparoscopic or open surgery) The extent of the surgery (e.g., gastric bypass vs. gastric sleeve) The physical demands of your job Any complications or adverse reactions to the surgery It is also important to remember that everyone recovers at their own pace, and it is normal to have some ups and downs during the recovery process. It is important to listen to your body and take breaks as needed to ensure that you are able to fully recover and return to your normal activities. | Similar | 1 | 1 | N/A |
| Will hair fall off after weight loss surgery? | It is common for people to experience some temporary hair loss after weight loss surgery. This can occur due to the stress of the surgery and the changes to your diet and nutrition that occur afterwards. Hair loss after weight loss surgery is typically temporary and should resolve on its own within a few months.  There are a few things you can do to help minimize hair loss after weight loss surgery:  Eat a well-balanced diet that includes a variety of nutrients, including protein, iron, and other minerals that are important for healthy hair growth. Avoid tight hairstyles, such as ponytails, that put excessive tension on the hair. Use a mild shampoo and conditioner and avoid styling products that may be harsh on the hair. Avoid using hot tools, such as flat irons and curling irons, on your hair. Consider using a hair growth supplement or using essential oils, such as rosemary oil or peppermint oil, to help stimulate hair growth. If you are concerned about hair loss after weight loss surgery, it is important to talk to your healthcare provider for guidance and support. They can help you identify any underlying causes and recommend treatment options if necessary. | Similar | 1 | 1 | N/A |
| Is plastic surgery necessary after weight loss surgery? | Plastic surgery is not necessarily required after weight loss surgery, but it can be an option for people who want to address excess skin or other physical changes that can occur after significant weight loss. It is common for people to have excess skin after weight loss surgery, especially if they have lost a large amount of weight in a short period of time. This excess skin can be uncomfortable and may cause physical issues such as rashes or infections.  If you are considering plastic surgery after weight loss surgery, it is important to talk to your healthcare provider and a plastic surgeon to determine if it is appropriate for you and to understand the potential risks and benefits. They can help you decide if plastic surgery is the right option for you and, if so, which procedures may be most suitable. It is also important to have realistic expectations about the outcome of plastic surgery and to understand that it may not be possible to achieve your desired results in a single procedure. Multiple procedures may be needed to achieve the desired outcome. | Similar | 1 | 1 | N/A |
| Do you have to take vitamins after weight loss surgery? | It is generally recommended for people who have had weight loss surgery to take certain vitamins and minerals to help prevent deficiencies and support their overall health. The specific vitamin and mineral supplements that are recommended after weight loss surgery may vary depending on the type of surgery you had and your individual needs.  In general, it is recommended that people who have had weight loss surgery take the following supplements:  A multi-vitamin that includes at least 100% of the recommended daily value of vitamins and minerals Vitamin B12 Calcium Iron It is important to follow the supplement recommendations provided by your healthcare team and to take your vitamins as directed. It is also important to eat a well-balanced diet that includes a variety of nutrient-dense foods to help ensure that you are getting all of the nutrients you need. If you have any concerns about your nutrient intake or are having difficulty taking your supplements as prescribed, it is important to talk to your healthcare provider for guidance. They can help you determine the best approach for meeting your nutritional needs. | Similar | 1 | 1 | N/A |
| Do weight loss surgery patients have serious health problems caused by vitamin deficiency? | Weight loss surgery can increase the risk of certain nutrient deficiencies, particularly in the first few months after the procedure. This is because weight loss surgery can alter the way the body absorbs nutrients from food. It is important for people who have had weight loss surgery to take vitamin and mineral supplements as recommended by their healthcare team and to eat a well-balanced diet that includes a variety of nutrient-dense foods to help prevent deficiencies and support their overall health.  If nutrient deficiencies are not properly managed, they can potentially lead to serious health problems. For example, a deficiency in iron can cause anemia, which can lead to fatigue and difficulty concentrating. A deficiency in vitamin B12 can cause nerve damage and affect the production of red blood cells. A deficiency in calcium can weaken the bones and increase the risk of osteoporosis. It is important to monitor your nutrient levels regularly and to take the necessary steps to ensure that you are getting all of the nutrients you need to support your overall health. If you have any concerns about your nutrient intake or are experiencing any unusual symptoms, it is important to talk to your healthcare provider for guidance. | Similar | 1 | 1 | N/A |
| Does weight loss surgery increase the risk for alcohol abuse? | There is some evidence to suggest that weight loss surgery may be associated with an increased risk of alcohol abuse. One study found that people who had weight loss surgery were more likely to report binge drinking and alcohol use disorders compared to those who did not have the surgery. It is not clear why this may be the case, but some researchers believe that weight loss surgery may alter the way the body processes alcohol, making it more intoxicating and potentially leading to increased alcohol consumption.  It is important to note that weight loss surgery is not a guarantee of weight loss or improved health, and it is not a substitute for healthy lifestyle behaviors such as maintaining a healthy diet and avoiding excessive alcohol intake. If you are considering weight loss surgery, it is important to discuss any concerns about alcohol use with your healthcare provider and to follow their recommendations for managing your alcohol intake after surgery. It is also important to be aware of the potential risks of alcohol abuse and to seek help if you are struggling with alcohol dependency. | There is some evidence to suggest that weight loss surgery may be associated with an increased risk of alcohol abuse, especially in people who have a history of alcohol use disorder (AUD) or other substance abuse problems.  One study found that people who had weight loss surgery were more likely to develop AUD after surgery than those who did not have surgery. Another study found that people who had weight loss surgery were more likely to report heavy alcohol use after surgery, compared to those who did not have surgery.  It is not clear why weight loss surgery may be associated with an increased risk of alcohol abuse, but it is thought that the changes in the body's metabolism and brain chemistry that occur after surgery may play a role.  It is important to remember that weight loss surgery is a serious and often life-changing treatment, and it is not a guarantee of long-term weight loss or improved health. If you are considering weight loss surgery, it is important to discuss any history of alcohol use or substance abuse with your healthcare team. They can help you understand the potential risks and benefits and provide guidance on how to optimize your health and well-being before and after surgery. | 1 | 2,1 | 1 |
| Does weight loss surgery increase the risk for suicide? | There is some evidence to suggest that weight loss surgery may be associated with an increased risk of suicide. One study found that people who had weight loss surgery were more likely to die by suicide compared to those who did not have the surgery. However, it is not clear why this may be the case and more research is needed to understand the relationship between weight loss surgery and suicide risk.  It is important to note that weight loss surgery is not a guarantee of weight loss or improved health, and it is not a substitute for healthy lifestyle behaviors such as maintaining a healthy diet and engaging in regular physical activity. If you are considering weight loss surgery, it is important to discuss any concerns about your mental health with your healthcare provider and to seek help if you are struggling with thoughts of suicide or other mental health issues. It is also important to be aware of the potential risks associated with weight loss surgery and to carefully weigh the potential benefits and risks before deciding to undergo the procedure. | There is some evidence to suggest that weight loss surgery may be associated with an increased risk of suicide, although the evidence is limited and the exact risk is not known.  One study found that people who had weight loss surgery were more likely to report suicidal thoughts and behaviors after surgery, compared to those who did not have surgery. Another study found that people who had weight loss surgery were more likely to die by suicide after surgery, compared to those who did not have surgery.  It is not clear why weight loss surgery may be associated with an increased risk of suicide, but it is thought that the psychological and emotional changes that can occur after surgery may play a role. Some people may experience changes in mood, body image, and social relationships after weight loss surgery, which may contribute to an increased risk of suicide.  If you are considering weight loss surgery, it is important to discuss any history of mental health issues with your healthcare team. They can help you understand the potential risks and benefits and provide guidance on how to optimize your health and well-being before and after surgery. It is also important to seek support and treatment if you are experiencing suicidal thoughts or behaviors. You can talk to your healthcare team or a mental health professional, or you can call a crisis hotline for help. | 2, 1 | 2, 1 | N/A |
| Is the chance of dying from weight loss surgery more than the chance of dying from obesity? | Weight loss surgery is generally considered to be safe and effective, but like any surgery, it carries certain risks. The risk of complications and death from weight loss surgery is generally low, but it is higher than the risk of not having surgery. The specific risks of weight loss surgery may vary depending on the type of surgery you have and your individual circumstances.  Obesity itself carries certain health risks, including an increased risk of certain diseases such as type 2 diabetes, heart disease, and certain cancers. While the risks of obesity are significant, the risk of death from obesity is generally lower than the risk of death from weight loss surgery.  It is important to carefully consider the potential risks and benefits of weight loss surgery and to discuss your options with your healthcare provider before making a decision. They can help you determine the best approach for managing your weight and optimizing your overall health. | Weight loss surgery is a safe and effective treatment for obesity and related conditions, and it can help people lose a significant amount of weight and improve their overall health. However, like any surgery, weight loss surgery carries some risks, including the risk of complications and death.  The risk of complications and death after weight loss surgery varies depending on the type of surgery, the individual's overall health, and other factors. In general, the risks of weight loss surgery are low, and the benefits of surgery typically outweigh the risks.  However, it is important to remember that obesity itself is a serious and potentially life-threatening condition, and it is associated with an increased risk of a number of health problems, including heart disease, stroke, type 2 diabetes, and certain types of cancer. For some people with severe obesity, the risks of obesity may be higher than the risks of weight loss surgery.  It is important to carefully consider the risks and benefits of weight loss surgery and to discuss your options with a healthcare team before making a decision. They can help you understand the potential risks and benefits and provide guidance on the best treatment plan for your individual needs and goals. | 3, 2 | 3, 1 | 3, 3 |
| What can you do with the extra skin after weight loss surgery? | After significant weight loss, it is common for people to have excess skin that cannot be eliminated through diet and exercise alone. This excess skin can be uncomfortable and may cause physical issues such as rashes or infections. If you are concerned about excess skin after weight loss surgery, you may be a candidate for plastic surgery to remove the excess skin.  Plastic surgery procedures that may be used to address excess skin after weight loss surgery include:  Tummy tuck (abdominoplasty) to remove excess skin from the abdomen Breast lift (mastopexy) to lift and reshape the breasts Arm lift (brachioplasty) to remove excess skin from the upper arms Thigh lift to remove excess skin from the thighs Facelift to tighten the skin on the face and neck If you are considering plastic surgery to address excess skin after weight loss surgery, it is important to talk to a plastic surgeon and your healthcare provider to determine if it is appropriate for you and to understand the potential risks and benefits. They can help you decide if plastic surgery is the right option for you and, if so, which procedures may be most suitable. It is also important to have realistic expectations about the outcome of plastic surgery and to understand that it may not be possible to achieve your desired results in a single procedure. Multiple procedures may be needed to achieve the desired outcome. | Similar | 1 | 1 | N/A |
| What are the scars like for each type of weight loss surgery? | The scars from weight loss surgery can vary depending on the type of surgery you have. Here is a general overview of the scars associated with each type of weight loss surgery:  Gastric bypass surgery: This procedure typically involves making five to six small incisions in the abdomen. The scars from these incisions may be visible and may take several months to a year to fade.  Sleeve gastrectomy: This procedure involves making a single incision down the middle of the abdomen. The scar from this incision may be visible and may take several months to a year to fade.  Adjustable gastric band: This procedure involves making a small incision in the abdomen and another incision in the chest. The scars from these incisions may be visible and may take several months to a year to fade.  Biliopancreatic diversion with duodenal switch: This procedure typically involves making a single incision down the middle of the abdomen. The scar from this incision may be visible and may take several months to a year to fade.  It is important to keep in mind that everyone heals differently and the appearance of scars can vary. It is important to follow the scar care instructions provided by your healthcare team to help minimize scarring and optimize the healing process. If you are concerned about the appearance of scars, it is important to talk to your healthcare provider for guidance. They can provide recommendations for scar care and may be able | There are several types of weight loss surgery, also known as bariatric surgery, and each one has its own unique scar. Here is a brief overview of the most common types of weight loss surgery and their corresponding scars:  Gastric bypass: This procedure involves creating a small pouch at the top of the stomach and attaching it directly to the small intestine. The scar is typically about 5-6 inches long and is located on the lower abdomen.  Sleeve gastrectomy: This procedure involves removing a large portion of the stomach, leaving a narrow "sleeve" shape. The scar is typically about 5-6 inches long and is located on the upper part of the abdomen.  Gastric band: This procedure involves placing an inflatable band around the upper part of the stomach to create a small pouch. The scar is usually small and is located on the upper abdomen.  Duodenal switch: This procedure involves removing a large portion of the stomach and reattaching the small intestine to the remaining part of the stomach. The scar is typically about 6-8 inches long and is located on the lower abdomen.  It's important to note that the appearance and size of the scar can vary depending on the individual patient and the specific technique used by the surgeon. In general, weight loss surgery scars tend to fade over time and become less noticeable. | 4 | 4, 4 | N/A |
| Is there a difference in how much pain afterward depending on which kind of weight loss surgery? | The amount of pain after weight loss surgery can vary depending on the type of surgery you have and your individual pain tolerance. In general, gastric bypass surgery and biliopancreatic diversion with duodenal switch may be associated with more pain than sleeve gastrectomy or adjustable gastric band surgery.  It is common to experience some pain and discomfort after weight loss surgery, but this can usually be managed with pain medication. Your healthcare team will provide you with pain management guidelines and medication as needed to help you manage your pain.  It is important to follow your healthcare team's instructions for pain management and to let them know if you are experiencing significant pain or discomfort. They can help you determine the cause of the pain and adjust your pain management plan as needed. It is also important to keep in mind that everyone's experience with pain after weight loss surgery is different, and what is tolerable for one person may not be tolerable for another. It is important to communicate with your healthcare team about your pain and to follow their recommendations for managing it. | The amount of pain experienced after weight loss surgery can vary depending on the type of procedure, the individual patient, and their pain tolerance. In general, most patients experience some pain after weight loss surgery, but the severity of the pain can vary.  Gastric bypass and duodenal switch procedures tend to be more invasive and may be associated with more pain than less invasive procedures such as a gastric band or sleeve gastrectomy. However, every patient is different, and some people may experience more or less pain with one type of procedure compared to another.  Pain after weight loss surgery is usually managed with a combination of pain medication and other techniques such as ice or heat therapy. Most patients are able to return to their normal activities within a few weeks, although the exact timeline can vary depending on the individual. It's important to follow the instructions of the healthcare team and report any persistent or severe pain to the doctor. | 1 | 3,3 | 3 |
| Is there a difference in how long it will take to get better after depending on which type of weight loss surgery? | The length of time it takes to recover after weight loss surgery can vary depending on the type of surgery you have and your individual circumstances. In general, sleeve gastrectomy and adjustable gastric band surgery may have shorter recovery times compared to gastric bypass surgery and biliopancreatic diversion with duodenal switch.  It is important to follow your healthcare team's instructions for post-surgery care and to allow yourself sufficient time to rest and heal. It is also important to be patient and to remember that everyone's recovery is different. It may take several weeks or months to fully recover from weight loss surgery and to feel back to your normal self.  It is important to keep in mind that weight loss surgery is a major procedure and it is important to take the necessary time to recover properly. This may involve taking time off work, avoiding strenuous activities, and following your healthcare team's instructions for post-surgery care. By following these guidelines, you can help ensure a smooth recovery and optimize your chances of success after surgery. | The length of time it takes to recover from weight loss surgery can vary depending on the type of procedure, the individual patient, and their overall health. In general, gastric bypass and duodenal switch procedures are more invasive and may be associated with a longer recovery time compared to less invasive procedures such as a gastric band or sleeve gastrectomy.  Most patients are able to return to their normal activities within a few weeks after weight loss surgery, although the exact timeline can vary. It's important to follow the instructions of the healthcare team and take steps to ensure a smooth recovery, such as getting enough rest, following a healthy diet, and avoiding heavy lifting or strenuous activities.  It's also important to note that weight loss surgery is a major procedure that requires a long-term commitment to lifestyle changes, such as following a healthy diet and getting regular exercise. These changes can help to ensure the best possible outcome and long-term success after weight loss surgery. | 1 | 3, 3 | 3 |
| How often do you need to see the doctor during the first year after weight loss surgery? | It is important for patients to have regular follow-up visits with their healthcare team after weight loss surgery. The frequency of these visits may vary depending on the type of surgery and the patient's individual needs, but it is common for patients to see the healthcare team every few weeks or months during the first year after surgery.  During these visits, the healthcare team will monitor the patient's progress and address any concerns or complications that may arise. The patient may also receive support and guidance on lifestyle changes, such as diet and exercise, to help them maintain their weight loss and improve their overall health.  It is important for patients to attend all follow-up visits as recommended by their healthcare team, as these visits are an important part of the recovery process and can help ensure the best possible outcome after surgery. | Similar | 1 | 1 | N/A |
| How long after weight loss surgery can you have caffeine? | It is generally recommended to avoid caffeine for the first two to three weeks after weight loss surgery, as caffeine can interfere with the healing process and cause digestive symptoms such as stomach cramps, diarrhea, and bloating.  After this initial period, most patients can slowly reintroduce caffeine into their diet. However, it is important to consume caffeine in moderation, as it can have a diuretic effect and may contribute to dehydration. It is also a good idea to avoid consuming caffeine in large amounts or immediately before or after meals, as it can interfere with the absorption of nutrients.  It is important to follow the guidelines provided by your healthcare team and to listen to your body's cues. If you experience any adverse effects after consuming caffeine, you may need to limit your intake or avoid it altogether. Your healthcare team can provide more specific recommendations based on your individual circumstances and the type of weight loss surgery you have undergone. | Similar | 1 | 1 | N/A |
| What can you eat 2 weeks after weight loss surgery? | The types of foods that you can eat after weight loss surgery will depend on the specific procedure you have undergone and your individual recovery. In general, it is important to follow the dietary guidelines provided by your healthcare team after surgery, as your body will be adjusting to significant changes in your digestive system.  During the first two weeks after weight loss surgery, you will likely be on a clear liquids diet, which may include water, broth, and sugar-free drinks. You may also be able to have very small amounts of low-fat, high-protein foods, such as eggs, chicken, or fish. It is important to consume only small amounts of food at a time and to chew your food well to help prevent digestive symptoms such as nausea, vomiting, and bloating.  As you progress through the recovery process, your healthcare team will provide you with guidelines on how to gradually introduce solid foods back into your diet. This may involve starting with pureed or soft foods and gradually transitioning to more solid foods as tolerated. It is important to follow these guidelines carefully and to listen to your body's cues to help ensure a smooth recovery and avoid digestive symptoms. | After weight loss surgery, you will need to follow a special diet to help your stomach heal and to ensure that you get enough nutrients. The specific diet that you will need to follow will depend on the type of weight loss surgery that you had and the specific instructions of your surgeon.  In general, during the first two weeks after weight loss surgery, you will need to follow a liquid diet. This may include clear liquids such as water, broth, and sugar-free drinks, as well as pureed or soft foods such as yogurt, pureed vegetables, and protein shakes.  It's important to follow the instructions of your surgeon and healthcare team and to consume small, frequent meals to help your stomach heal. You may also need to take vitamin and mineral supplements as directed by your surgeon to ensure that you are getting enough nutrients.  As you progress through the post-surgery diet, you will typically be able to gradually add more solid foods back into your diet. It's important to listen to your body and to eat slowly, taking small bites and chewing your food thoroughly to avoid discomfort. | 1 | 3, 1 | 3, 1 |
| How soon after weight loss surgery can you travel? | The timing of when you can travel after weight loss surgery will depend on the specific procedure you have undergone and your individual recovery. It is important to discuss your travel plans with your healthcare team and to follow their recommendations.  In general, it is recommended to wait at least two to three weeks after weight loss surgery before traveling. This allows time for the body to heal and recover from the surgery. During this time, it is important to follow the guidelines provided by your healthcare team for diet, activity, and medications.  If you do need to travel within the first few weeks after surgery, it is important to plan ahead and make arrangements for any additional support or medical care you may need while away from home. It is also a good idea to carry a copy of your medical records, a list of your medications, and the contact information for your healthcare team in case of an emergency.  As you progress through the recovery process, your healthcare team will provide you with guidance on when it is safe to resume normal activities, including travel. It is important to follow these guidelines carefully and to listen to your body's cues to help ensure a smooth recovery. | Similar | 1 | 1 | N/A |
| How long after weight loss surgery does nausea go away? | Nausea is a common side effect after weight loss surgery, and the timing of when it resolves can vary depending on the individual patient and the type of surgery they have undergone.  In general, nausea after weight loss surgery tends to resolve within a few days to a week. However, some patients may continue to experience nausea for a longer period of time, particularly if they are not following the dietary guidelines provided by their healthcare team or if they are experiencing other complications such as dehydration or an infection.  It is important to follow the guidelines provided by your healthcare team to help manage nausea and other side effects after weight loss surgery. This may include taking prescribed medications as directed, eating small, frequent meals, and avoiding foods that are high in fat or sugar. If you are experiencing persistent or severe nausea, it is important to contact your healthcare team for further evaluation and treatment. | Similar | 1 | 1 | N/A |
| What can you eat on a day-to-day basis after weight loss surgery after finishing the liquid diet? | After you have completed the clear liquids diet following weight loss surgery, your healthcare team will provide you with guidelines on how to gradually introduce solid foods back into your diet. This process may involve starting with pureed or soft foods and gradually transitioning to more solid foods as tolerated.  It is important to follow the guidelines provided by your healthcare team carefully and to listen to your body's cues to help ensure a smooth recovery and avoid digestive symptoms such as nausea, vomiting, and bloating.  In general, it is recommended to focus on high-protein, low-fat, and nutrient-dense foods after weight loss surgery. This may include:  Lean meats, such as chicken, turkey, fish, and tofu Non-starchy vegetables, such as leafy greens, bell peppers, and tomatoes Fruits, such as berries, apples, and pears Whole grains, such as quinoa, oats, and brown rice Low-fat dairy products, such as milk, yogurt, and cheese It is important to consume small, frequent meals and to chew your food well after weight loss surgery to help prevent digestive symptoms. You should also drink plenty of fluids, as it is important to stay hydrated after surgery.  It is also a good idea to avoid foods that are high in fat or sugar, as these can interfere with weight loss and increase the risk of digestive symptoms. Your healthcare team can provide more specific recommendations based on your individual needs and the type of weight loss surgery you have undergone. | Similar | 1 | 1 | N/A |
| How long after weight loss surgery can you drink alcohol? | It is generally recommended to avoid alcohol for the first two to three weeks after weight loss surgery, as alcohol can interfere with the healing process and cause digestive symptoms such as stomach cramps, diarrhea, and bloating.  After this initial period, most patients can slowly reintroduce alcohol into their diet. However, it is important to consume alcohol in moderation, as it can have a dehydrating effect and may interfere with the absorption of nutrients. It is also a good idea to avoid consuming alcohol in large amounts or immediately before or after meals, as it can interfere with the digestion of food.  It is important to follow the guidelines provided by your healthcare team and to listen to your body's cues. If you experience any adverse effects after consuming alcohol, you may need to limit your intake or avoid it altogether. Your healthcare team can provide more specific recommendations based on your individual circumstances and the type of weight loss surgery you have undergone. | Similar | 2 | 2 | N/A |
| How much carbohydrates can you consume each day after weight loss surgery? | The amount of carbohydrates that you can consume after weight loss surgery will depend on the specific procedure you have undergone and your individual needs and goals. It is important to follow the guidelines provided by your healthcare team and to listen to your body's cues to help ensure a healthy and balanced diet.  In general, it is recommended to focus on high-quality carbohydrates that are rich in fiber and nutrients after weight loss surgery. These may include whole grains, vegetables, and fruits. It is important to choose foods that are low in added sugars and to avoid highly processed or refined carbohydrates, such as white bread, pastries, and sugary drinks.  It is also a good idea to pay attention to portion sizes and to consume carbohydrates in moderation after weight loss surgery. Your healthcare team can provide more specific recommendations based on your individual needs and goals.  It is important to remember that the goal of weight loss surgery is not to completely eliminate carbohydrates from the diet, but rather to help the patient make healthier food choices and achieve a healthy weight. A healthy diet after weight loss surgery should include a variety of nutrients and be balanced to support overall health and well-being. | Similar | 1 | 1 | N/A |
| What stool softener is recommended after weight loss surgery? | Stool softeners may be recommended after weight loss surgery to help prevent constipation and promote regular bowel movements. It is important to follow the recommendations of your healthcare team when choosing a stool softener, as different products may be more or less appropriate depending on your individual needs and medical history.  Some common stool softeners that may be recommended after weight loss surgery include:  Docusate (Colace) Sennosides (Senokot) Psyllium (Metamucil) It is important to follow the instructions provided by the manufacturer and to use the product as directed. If you experience any adverse effects or if the product does not seem to be effective, it is important to contact your healthcare team for further evaluation and guidance.  It is also a good idea to drink plenty of fluids and to include fiber-rich foods in your diet after weight loss surgery to help promote regular bowel movements. Your healthcare team can provide more specific recommendations based on your individual needs and the type of weight loss surgery you have undergone. | It is generally recommended to avoid using stool softeners after weight loss surgery, as they can interfere with the absorption of nutrients and lead to deficiencies. Instead, it is often recommended to increase fluid and fiber intake to help promote regular bowel movements. Some high-fiber foods that may be helpful include fruits, vegetables, whole grains, and legumes. If you are having difficulty with constipation, it is important to talk to your doctor or a registered dietitian for personalized recommendations. | 1, 3 | 1, 3 | N/A |
| Will there be any extra complications from weight loss surgery for someone with an autoimmune disorder? | It is possible that individuals with autoimmune disorders may be at an increased risk of certain complications after weight loss surgery. This is because autoimmune disorders can affect the body's ability to absorb nutrients and may make the body more susceptible to certain complications, such as malnutrition or infections.  It is important to discuss the potential risks and benefits of weight loss surgery with your healthcare team and to carefully consider the potential impacts on your autoimmune disorder. Your healthcare team can help you make an informed decision about whether weight loss surgery is right for you and can provide you with guidance on how to manage your condition before and after surgery.  If you do decide to undergo weight loss surgery, it is important to follow the guidelines provided by your healthcare team and to attend all follow-up visits as recommended. It is also a good idea to communicate with your healthcare team about any changes in your health or any symptoms that you are experiencing. This can help to identify and address any potential complications early on and improve your chances of a successful outcome. | Similar | 1 | 1 | N/A |
| Can weight loss surgery trigger an autoimmune disease? | There is no evidence to suggest that weight loss surgery can trigger an autoimmune disease. Autoimmune diseases are complex conditions that are thought to be caused by a combination of genetic, environmental, and other factors. Weight loss surgery does not directly cause autoimmune diseases.  However, it is possible that the physical and emotional changes that can occur after weight loss surgery may affect the course or management of an autoimmune disease. For example, rapid weight loss or changes in the body's metabolism may affect the way the body responds to medications or may cause other physiological changes. It is important to discuss the potential impacts of weight loss surgery on your autoimmune disease with your healthcare team and to carefully consider the potential risks and benefits of the procedure.  It is also important to continue to manage your autoimmune disease as prescribed by your healthcare team after weight loss surgery. This may include taking medications as directed, following a healthy diet and lifestyle, and attending regular medical appointments. Your healthcare team can provide you with guidance on how to manage your condition before and after surgery. | There is no evidence to suggest that weight loss surgery can directly cause or trigger autoimmune diseases. However, it is possible that individuals who undergo weight loss surgery may be at an increased risk of developing certain autoimmune conditions due to changes in their gut microbiota, which can occur as a result of the surgery. It is important for individuals to be aware of the potential risks and to closely monitor their health after weight loss surgery. If you are concerned about the potential risks of weight loss surgery, it is important to discuss your concerns with your healthcare team. They can help you understand the potential risks and benefits of the procedure and help you make an informed decision about whether weight loss surgery is right for you. | 1, 3 | 1 | 1, 3 |
| Is it normal to experience nausea and vomiting years after bariatric surgery? | It is not uncommon for patients to experience nausea and vomiting in the immediate aftermath of weight loss surgery, as the body adjusts to the changes in the digestive system. However, it is generally not normal to experience these symptoms years after surgery.  If you are experiencing persistent or recurrent nausea and vomiting after weight loss surgery, it is important to contact your healthcare team for further evaluation and treatment. There may be an underlying cause for these symptoms, such as a dietary intolerance, a gastrointestinal disorder, or a complication of the surgery. Your healthcare team can help identify the cause of your symptoms and provide you with appropriate treatment.  It is also important to follow the guidelines provided by your healthcare team for diet and lifestyle after weight loss surgery to help prevent digestive symptoms and ensure a healthy recovery. This may include eating small, frequent meals, avoiding foods that are high in fat or sugar, and drinking plenty of fluids. Your healthcare team can provide you with more specific recommendations based on your individual needs and the type of weight loss surgery you have undergone. | Similar | 1 | 1 | N/A |
| Is it normal to have lower back pain after bariatric surgery? | Lower back pain is a common side effect after weight loss surgery, particularly in the first few weeks or months after the procedure. This is often due to the physical changes that occur as a result of the surgery, such as rapid weight loss or changes in posture.  It is generally recommended to follow the guidelines provided by your healthcare team for activity and exercise after weight loss surgery to help reduce the risk of back pain and other complications. This may include taking frequent breaks to rest and stretch, avoiding activities that put strain on the back, and avoiding lifting heavy objects. Your healthcare team can provide you with more specific recommendations based on your individual needs and the type of weight loss surgery you have undergone.  If you are experiencing persistent or severe lower back pain after weight loss surgery, it is important to contact your healthcare team for further evaluation and treatment. There may be an underlying cause for your pain, such as a muscle strain or a spinal issue, and your healthcare team can help identify the cause and provide you with appropriate treatment. | Similar | 1 | 1 | N/A |
| What is recommended for the liquid diet after bariatric surgery? | After weight loss surgery, it is generally recommended to follow a clear liquids diet for the first few days to help the body adjust to the changes in the digestive system. A clear liquids diet may include:  Water Broth Sugar-free drinks, such as coffee, tea, and clear soda Electrolyte solutions, such as Gatorade or Pedialyte It is important to follow the guidelines provided by your healthcare team carefully and to consume only the liquids that are recommended. It is also important to drink plenty of fluids to help stay hydrated and support the healing process.  After a few days on a clear liquids diet, your healthcare team may recommend transitioning to a full liquids diet, which may include non-clear liquids such as milk, cream, and pureed soups. They may then recommend gradually introducing solid foods back into your diet as tolerated.  It is important to follow the guidelines provided by your healthcare team carefully and to listen to your body's cues to help ensure a smooth recovery and avoid digestive symptoms such as nausea, vomiting, and bloating. Your healthcare team can provide you with more specific recommendations based on your individual needs and the type of weight loss surgery you have undergone. | Similar | 1 | 1 | N/A |
| When does the burning pain from the incisions go away after bariatric surgery? | The burning pain from the incisions after weight loss surgery may resolve within a few days to a week after the procedure. However, the timing can vary depending on the individual patient and the type of surgery they have undergone.  It is generally recommended to follow the guidelines provided by your healthcare team for pain management after weight loss surgery to help reduce the risk of complications and promote a smooth recovery. This may include taking prescribed pain medications as directed and avoiding activities that put strain on the incisions.  If you are experiencing persistent or severe burning pain from the incisions after weight loss surgery, it is important to contact your healthcare team for further evaluation and treatment. They can help identify the cause of your pain and provide you with appropriate treatment.  It is also important to communicate with your healthcare team about any changes in your symptoms or any other concerns you may have after weight loss surgery. This can help to identify and address any potential complications early on and improve your chances of a successful outcome. | Similar | 1 | 1 | N/A |
| Does weight loss surgery cause hair loss? | Weight loss surgery can cause temporary hair loss in some patients, particularly in the first few months after the procedure. This is often due to the physical and hormonal changes that occur as a result of the surgery, such as rapid weight loss or changes in the body's metabolism.  It is generally recommended to follow the guidelines provided by your healthcare team for diet and nutrition after weight loss surgery to help reduce the risk of hair loss and other complications. This may include consuming a balanced diet that is rich in protein, vitamins, and minerals, and taking supplements as recommended.  If you are experiencing hair loss after weight loss surgery, it is important to communicate with your healthcare team about your concerns. They can help identify the cause of your hair loss and provide you with appropriate treatment. In most cases, hair loss after weight loss surgery is temporary and will resolve on its own over time.  It is also important to be patient and to give your body time to adjust to the changes that have occurred as a result of the surgery. It may take several months to a year for your hair to fully grow back after weight loss surgery. | Similar | 1 | 1 | N/A |
| Why would someone feel dizzy months after bariatric surgery? | There are several potential reasons why someone may feel dizzy months after weight loss surgery. Some possible causes of dizziness after weight loss surgery may include:  Dehydration: It is important to drink plenty of fluids after weight loss surgery to help stay hydrated and support the healing process. If you are not getting enough fluids, you may feel dizzy.  Nutrient deficiencies: Weight loss surgery can affect the body's ability to absorb nutrients, which can lead to deficiencies in certain vitamins and minerals. These deficiencies can cause symptoms such as dizziness.  Anemia: Anemia, or a low red blood cell count, can cause dizziness. Weight loss surgery can increase the risk of anemia, particularly in individuals who are already at risk for this condition.  Inner ear disorders: Dizziness can also be caused by inner ear disorders, such as vestibular neuritis or Meniere's disease. These disorders can be triggered by weight loss surgery or may be unrelated.  If you are experiencing dizziness after weight loss surgery, it is important to contact your healthcare team for further evaluation and treatment. They can help identify the cause of your symptoms and provide you with appropriate treatment. | Similar | 1 | 1 | N/A |
| How common are complications after weight loss surgery? | Complications after weight loss surgery are generally rare, but they can occur. The specific risks of complications will depend on the type of surgery you have undergone, your individual health and medical history, and other factors.  Some common complications after weight loss surgery may include:  Nausea, vomiting, and diarrhea Infection Bleeding Blood clots Nutrient deficiencies Anemia Gallstones Hernias Leakage from the surgical site It is important to carefully consider the potential risks and benefits of weight loss surgery and to discuss the potential complications with your healthcare team before making a decision about whether to undergo the procedure. Your healthcare team can provide you with more specific information about the risks and benefits of weight loss surgery based on your individual needs.  If you do decide to undergo weight loss surgery, it is important to follow the guidelines provided by your healthcare team to help reduce the risk of complications and promote a smooth recovery. This may include taking medications as prescribed, attending follow-up visits, and following a healthy diet and lifestyle. It is also important to communicate with your healthcare team about any changes in your health or any symptoms that you are experiencing after surgery. This can help to identify and address any potential complications early on and improve your chances of a successful outcome. | Weight loss surgery is generally safe, but as with any surgical procedure, there is a risk of complications. The specific risks of complications will depend on the type of surgery being performed and the individual's overall health. Some common complications after weight loss surgery include:  Infection: Infections can occur at the incision site or elsewhere in the body after surgery.  Blood clots: Blood clots can form in the legs or lungs after surgery, which can be serious if left untreated.  Nutrient deficiencies: Weight loss surgery can affect the body's ability to absorb certain nutrients, leading to deficiencies.  Dumping syndrome: This is a condition that occurs when food moves too quickly through the small intestine, causing symptoms such as nausea, vomiting, and dizziness.  Hernias: Hernias, or protrusion of abdominal tissue through a weak spot in the abdominal wall, can occur after weight loss surgery.  In general, the risk of complications after weight loss surgery is low, and most individuals experience a successful outcome. It is important to closely follow your healthcare team's instructions for post-surgery care to help reduce the risk of complications. If you experience any unusual symptoms or concerns after weight loss surgery, it is important to speak with your healthcare provider as soon as possible. | 1 | 2, 1 | 3, 2 |
| Will weight loss surgery cause lactose intolerance? | Weight loss surgery can increase the risk of lactose intolerance, a condition in which the body is unable to digest lactose, a sugar found in milk and milk products. This is because weight loss surgery can affect the body's ability to produce lactase, an enzyme that helps to break down lactose in the digestive system.  The risk of developing lactose intolerance after weight loss surgery may depend on the type of surgery you have undergone, as well as your individual health and medical history. Some types of weight loss surgery, such as the gastric bypass, may be more likely to cause lactose intolerance than others.  If you are experiencing symptoms of lactose intolerance after weight loss surgery, such as bloating, gas, diarrhea, or abdominal pain after consuming milk or milk products, it is important to contact your healthcare team for further evaluation and treatment. They can help determine the cause of your symptoms and provide you with appropriate treatment.  It is generally recommended to follow the guidelines provided by your healthcare team for diet and nutrition after weight loss surgery to help manage lactose intolerance and other digestive symptoms. This may include avoiding or limiting milk and milk products in your diet, or taking lactase supplements to help break down lactose. Your healthcare team can provide you with more specific recommendations based on your individual needs and the type of weight loss surgery you have undergone. | Weight loss surgery, also known as bariatric surgery, can cause lactose intolerance in some people. This is because the surgery can alter the structure and function of the small intestine, which can affect the body's ability to digest lactose.  However, it's important to note that not all people who have weight loss surgery will develop lactose intolerance. Some people may be more prone to developing lactose intolerance after surgery due to their genetics or other factors.  If you are considering weight loss surgery and are concerned about the potential for developing lactose intolerance, it's a good idea to talk to your doctor about your options and any potential risks and benefits. | 1, 2 | 1 | 1 |
| Is it normal to have a lot of loose skin after weight loss surgery? | It is not uncommon for patients to have excess or loose skin after weight loss surgery, particularly if they have lost a significant amount of weight. The amount of loose skin that is present after weight loss surgery will depend on a number of factors, including the individual's starting weight, the amount of weight they have lost, their age, and their skin elasticity.  If you are concerned about excess or loose skin after weight loss surgery, it is important to communicate with your healthcare team about your concerns. They can provide you with guidance and support and can help you to explore options for addressing excess skin, such as skin removal surgery or other treatments.  It is generally recommended to follow the guidelines provided by your healthcare team for diet and lifestyle after weight loss surgery to help maintain your weight loss and promote a healthy recovery. This may include eating a balanced diet that is rich in protein, fiber, and nutrients, getting regular physical activity, and managing stress. Your healthcare team can provide you with more specific recommendations based on your individual needs and the type of weight loss surgery you have undergone. | Similar | 1 | 1 | N/A |
| Will bariatric surgery cause new allergies to develop? | Weight loss surgery is not generally thought to cause new allergies to develop. However, weight loss surgery can affect the body's immune system, which can affect the way the body responds to allergens.  In some cases, patients may experience changes in their allergy symptoms after weight loss surgery, such as an increase or decrease in the severity of their allergies. These changes may be due to a variety of factors, including changes in the body's metabolism or immune system function as a result of the surgery.  If you are experiencing changes in your allergy symptoms after weight loss surgery, it is important to communicate with your healthcare team about your concerns. They can help to identify the cause of your symptoms and provide you with appropriate treatment.  It is generally recommended to follow the guidelines provided by your healthcare team for diet and nutrition after weight loss surgery to help manage any changes in allergy symptoms and promote a healthy recovery. This may include avoiding or limiting certain foods or substances that you are allergic to, or taking medications as prescribed. Your healthcare team can provide you with more specific recommendations based on your individual needs and the type of weight loss surgery you have undergone. | It is not common for bariatric surgery (weight loss surgery) to cause new allergies to develop. Allergies are caused by the immune system's reaction to a substance that is normally harmless. Weight loss surgery does not directly affect the immune system, so it is unlikely to cause new allergies to develop.  However, it is possible that some people may experience changes in their digestion and absorption of nutrients after weight loss surgery, which could potentially affect their tolerance for certain foods. If you are concerned about the potential for developing new allergies after weight loss surgery, it's a good idea to talk to your doctor. They can assess your individual situation and provide guidance on any potential risks and benefits. | 2, 1 | 1 | 1 |
| Is constipation after weight loss surgery normal? | Constipation is a common side effect after weight loss surgery, particularly in the first few weeks to months after the procedure. This is often due to the physical and hormonal changes that occur as a result of the surgery, such as changes in the digestive system or the body's metabolism.  If you are experiencing constipation after weight loss surgery, it is important to communicate with your healthcare team about your symptoms. They can help to identify the cause of your constipation and provide you with appropriate treatment. Treatment options may include medications, lifestyle changes, or other interventions.  It is generally recommended to follow the guidelines provided by your healthcare team for diet and lifestyle after weight loss surgery to help manage constipation and promote a healthy recovery. This may include increasing your intake of fluids and fiber, getting regular physical activity, and managing stress. Your healthcare team can provide you with more specific recommendations based on your individual needs and the type of weight loss surgery you have undergone. | Similar | 1 | 1 | N/A |
| How many days do you stay in the hospital for after weight loss surgery? | The length of stay in the hospital after weight loss surgery can vary depending on the type of surgery you have undergone, your individual health and medical history, and other factors. In general, patients can expect to stay in the hospital for one to three days after weight loss surgery.  During this time, you will be closely monitored by your healthcare team to ensure that you are recovering well and to identify and address any potential complications. Your healthcare team will also provide you with instructions for caring for yourself at home and for managing any pain or other symptoms you may be experiencing after the surgery.  It is important to follow the instructions provided by your healthcare team after weight loss surgery to help ensure a smooth recovery and to reduce the risk of complications. This may include taking medications as prescribed, attending follow-up visits, and following a healthy diet and lifestyle. It is also important to communicate with your healthcare team about any changes in your health or any concerns you may have after surgery. This can help to identify and address any potential complications early on and improve your chances of a successful outcome. | Similar | 1 | 1 | N/A |
| How long are bariatric surgeries? | The length of a weight loss surgery can vary depending on the type of surgery being performed and other factors. In general, weight loss surgeries can take anywhere from one to several hours to complete.  The specific length of a weight loss surgery will depend on the complexity of the procedure and the individual patient's health and medical history. Your healthcare team can provide you with more specific information about the length of your weight loss surgery based on your individual needs.  It is important to carefully consider the potential risks and benefits of weight loss surgery and to discuss the details of the procedure with your healthcare team before making a decision about whether to undergo the surgery. Your healthcare team can provide you with more information about the specific risks and benefits of weight loss surgery based on your individual needs. | Similar | 1 | 1 | N/A |
| What can be taken to relieve gas after weight loss surgery? | Gas is a common side effect after weight loss surgery, and it can be caused by a variety of factors, such as changes in the digestive system or the body's metabolism. If you are experiencing gas after weight loss surgery, there are a few things that you can try to help relieve your symptoms:  Eat slowly and chew your food thoroughly: This can help to reduce the amount of air that you swallow, which can lead to gas.  Avoid carbonated beverages: Carbonated drinks can increase gas production in the digestive system.  Avoid or limit gas-producing foods: Some foods, such as beans, broccoli, cabbage, and onions, can increase gas production.  Take over-the-counter medications: There are a number of over-the-counter medications that can help to relieve gas, such as simethicone or activated charcoal.  Try peppermint tea: Peppermint tea can help to relax the muscles in the digestive system and may help to relieve gas.  It is generally recommended to follow the guidelines provided by your healthcare team for diet and lifestyle after weight loss surgery to help manage gas and promote a healthy recovery. Your healthcare team can provide you with more specific recommendations based on your individual needs and the type of weight loss surgery you have undergone. If you are experiencing persistent or severe gas after weight loss surgery, it is important to contact your healthcare team for further evaluation and treatment. | Similar | 1 | 1 | N/A |
| How long after bypass surgery can you eat soft or pureed food? | After bypass surgery, patients typically progress through a series of stages in their diet, starting with liquids and gradually advancing to solid foods. The specific timing for each stage of the diet will depend on the individual patient's health and medical history, as well as the type of bypass surgery they have undergone.  In general, patients can expect to start on a liquid diet after bypass surgery and then progress to pureed or soft foods as they are able to tolerate them. The length of time that patients will remain on a pureed or soft food diet will depend on their individual recovery and tolerance.  It is important to follow the guidelines provided by your healthcare team for diet and nutrition after bypass surgery to ensure a smooth and successful recovery. Your healthcare team can provide you with more specific recommendations based on your individual needs and the type of bypass surgery you have undergone. If you have any questions or concerns about your diet after bypass surgery, it is important to communicate with your healthcare team for guidance. | Similar | 1 | 1 | N/A |
| Is it normal to cough up blood after weight loss surgery? | Coughing up blood (also known as hemoptysis) is not a normal side effect after weight loss surgery, and it can be a sign of a serious complication. If you are coughing up blood after weight loss surgery, it is important to contact your healthcare team immediately for evaluation and treatment.  There are a variety of potential causes of hemoptysis after weight loss surgery, including infection, bleeding, or damage to the respiratory system. Your healthcare team will perform a thorough evaluation to determine the cause of your symptoms and provide you with appropriate treatment.  It is generally recommended to follow the guidelines provided by your healthcare team for diet and lifestyle after weight loss surgery to help promote a healthy recovery and reduce the risk of complications. This may include taking medications as prescribed, getting regular physical activity, and managing stress. If you are experiencing persistent or severe hemoptysis after weight loss surgery, it is important to contact your healthcare team for further evaluation and treatment. | Similar | 1 | 1 | N/A |
| Can weight loss surgery cause menopause? | Weight loss surgery can potentially affect hormone levels and reproductive function, but it is not likely to cause menopause. Menopause is a natural process that occurs when the ovaries stop producing estrogen and progesterone, leading to the end of menstrual periods and fertility. It typically occurs in women between the ages of 45 and 55.  Weight loss surgery, such as gastric bypass or sleeve gastrectomy, can cause significant weight loss, which can in turn affect hormone levels and reproductive function. Some studies have suggested that weight loss surgery may lead to irregular periods or amenorrhea (absence of menstrual periods) in women of reproductive age, but it is not likely to cause menopause.  If you are experiencing changes in your menstrual cycle or other menopause-like symptoms after weight loss surgery, it is important to talk to your healthcare provider. They can help determine the cause and suggest appropriate treatment options. | Similar | 1 | 1 | N/A |
| How long after weight loss surgery can you sleep on your side? | It is generally recommended to avoid sleeping on your side for at least the first six weeks after weight loss surgery. This is because sleeping on your side can put pressure on the incision site and potentially disrupt the healing process.  After six weeks, you should be able to sleep on your side if it is comfortable for you. However, it is still important to be mindful of the incision site and avoid putting too much pressure on it.  If you have any concerns about sleeping positions after weight loss surgery, it is best to ask your healthcare provider for guidance. They can provide specific recommendations based on your individual situation. | After weight loss surgery, it is generally recommended that you avoid sleeping on your stomach for the first few weeks to help prevent complications and ensure proper healing. Sleeping on your back is generally considered the safest position after surgery.  After the first few weeks, you may be able to sleep on your side, but it is important to check with your surgeon or healthcare provider for specific instructions. It is also important to use a firm and supportive mattress and pillows to help ensure proper body alignment and comfort while sleeping.  If you have any concerns or questions about sleeping positions after weight loss surgery, it is important to discuss them with your surgeon or healthcare provider. They can provide guidance on the safest and most comfortable positions for you to sleep in. | 1 | 2, 1 | 1 |
| Is it normal to have pulmonary embolisms weeks after sleeve gastrectomy? | Pulmonary embolism is a serious condition that occurs when a blood clot becomes lodged in the lungs and blocks the flow of blood. It can be life-threatening and requires immediate medical attention.  Pulmonary embolisms are rare after weight loss surgery, but they can occur. The risk of pulmonary embolism after sleeve gastrectomy is generally low, but it is not uncommon to experience some shortness of breath and chest discomfort after the surgery. These symptoms are usually mild and resolve within a few days to a week.  If you are experiencing shortness of breath or chest discomfort that is severe or persists beyond a week after your surgery, it is important to contact your healthcare provider right away. They can evaluate your symptoms and determine the appropriate course of treatment. | Pulmonary embolism (PE) is a serious and potentially life-threatening condition that occurs when a blood clot travels to the lungs and blocks a blood vessel. While PE can occur at any time, it is generally not considered normal to have a PE weeks after sleeve gastrectomy.  There are several risk factors that can increase the risk of PE after weight loss surgery, including obesity, smoking, and a history of blood clots. It is important to discuss these risk factors with your surgeon or healthcare provider before surgery so that they can take appropriate precautions to minimize the risk of PE.  If you are experiencing symptoms of PE, such as shortness of breath, chest pain, or rapid heart rate, it is important to seek medical attention immediately. PE is a serious and potentially life-threatening condition that requires prompt treatment. Your surgeon or healthcare provider will be able to determine the cause of your symptoms and recommend the appropriate treatment. | 1 | 2, 1 | 1, 2 |
| How long after weight loss surgery can you drive? | The amount of time it takes to recover from weight loss surgery and when you can safely drive again will vary depending on the type of surgery you had, your overall health, and other factors.  In general, it is generally recommended to avoid driving for at least the first week after weight loss surgery. This is because you will likely be taking pain medication and may be feeling tired or weak. It is also important to give your body time to heal and adjust to the surgery.  After the first week, you should be able to drive as long as you feel physically and mentally able to do so safely. It is important to follow the advice of your healthcare provider and only resume driving when you feel comfortable and confident in your ability to do so safely.  If you are unsure about when it is safe for you to drive after weight loss surgery, it is best to ask your healthcare provider for guidance. They can provide specific recommendations based on your individual situation. | Similar | 1 | 1 | N/A |
| Can weight loss surgery make GERD worse? | Weight loss surgery, such as gastric bypass or sleeve gastrectomy, can potentially affect the digestive system and may cause or worsen symptoms of gastroesophageal reflux disease (GERD).  GERD is a condition in which stomach acid flows back into the esophagus (the tube that connects the mouth to the stomach), causing heartburn and other symptoms. Weight loss surgery can cause changes in the digestive system that may lead to the development of GERD or make existing GERD worse.  If you have had weight loss surgery and are experiencing symptoms of GERD, such as heartburn or acid reflux, it is important to talk to your healthcare provider. They can evaluate your symptoms and suggest appropriate treatment options. In some cases, lifestyle changes, such as changes to your diet or sleep position, or medication may be recommended to manage GERD symptoms. | Similar | 1 | 1 | N/A |
| Why is there a limit on water consumption after weight loss surgery? | After weight loss surgery, it is important to limit fluid intake to avoid dehydration and to prevent complications such as dumping syndrome.  Dumping syndrome is a condition that can occur after weight loss surgery in which the contents of the stomach are emptied too quickly into the small intestine. This can cause symptoms such as nausea, vomiting, abdominal pain, and diarrhea.  To prevent dumping syndrome and ensure proper hydration, most weight loss surgery programs recommend limiting fluid intake to 8-12 ounces per day for the first few weeks after surgery. As you recover and your stomach and digestive system adjust to the surgery, your healthcare team will gradually increase your fluid intake.  It is important to follow your healthcare team's recommendations for fluid intake after weight loss surgery to ensure a safe and successful recovery. If you have any concerns about your fluid intake, it is best to ask your healthcare provider for guidance. | After weight loss surgery, it is generally recommended that you limit your water intake to help prevent complications such as dehydration and overhydration. In some cases, drinking too much water can lead to an imbalance of electrolytes in the body, which can cause symptoms such as fatigue, muscle cramps, and dizziness.  The specific recommendations for water intake after weight loss surgery may vary depending on the type of surgery you had and the recommendations of your surgeon or healthcare provider. In general, it is recommended that you drink small amounts of water throughout the day rather than trying to drink a large amount all at once. Sipping water slowly can help prevent symptoms such as nausea and bloating and can help ensure that your body is able to absorb the water properly.  It is important to follow the instructions of your surgeon or healthcare provider regarding water intake after weight loss surgery. They will be able to provide guidance based on your individual needs and can help ensure that you are getting enough fluids to stay hydrated while also avoiding complications. | 3, 2 | 3, 1 | 4,4 |
| Is it okay to take laxatives after weight loss surgery? | Laxatives are medications that are used to treat constipation or to stimulate bowel movements. While they can be helpful in certain situations, it is generally not recommended to use laxatives regularly after weight loss surgery.  After weight loss surgery, the digestive system may be slower and less efficient, which can lead to constipation. Constipation can be uncomfortable and may interfere with the healing process.  If you are experiencing constipation after weight loss surgery, it is important to talk to your healthcare provider. They can recommend safe and effective treatments to help alleviate your symptoms. In some cases, lifestyle changes, such as increasing your fluid and fiber intake or getting regular exercise, may be recommended to help relieve constipation.  Using laxatives regularly can lead to dependence and may interfere with the natural functioning of the digestive system. It is generally not recommended to use laxatives as a long-term solution for constipation. If you have any concerns about using laxatives after weight loss surgery, it is best to ask your healthcare provider for guidance. | Similar | 1 | 2 | 2 |
| Is it normal to feel dehydrated after weight loss surgery? | It is not uncommon to feel dehydrated after weight loss surgery. This can be due to a number of factors, including decreased fluid intake, increased fluid losses, and changes in the body's ability to absorb fluids.  After weight loss surgery, it is important to be mindful of your fluid intake and to drink enough fluids to stay hydrated. It is generally recommended to drink 8-12 ounces of fluids per day for the first few weeks after surgery, and then gradually increase your intake as recommended by your healthcare team.  If you are experiencing dehydration after weight loss surgery, it is important to talk to your healthcare provider. They can evaluate your symptoms and suggest appropriate treatment options, such as increasing your fluid intake or prescribing medications to help manage dehydration.  It is also important to remember that dehydration can have serious consequences and can lead to serious health problems if left untreated. If you are experiencing severe or persistent dehydration, it is important to seek medical attention right away. | It is not uncommon to feel dehydrated after weight loss surgery, particularly in the first few weeks after the procedure. This can be due to a number of factors, including the surgery itself, changes in your diet and fluid intake, and changes in your body's ability to absorb fluids.  After weight loss surgery, it is important to pay attention to your fluid intake and to make sure you are getting enough fluids to stay hydrated. It is generally recommended that you drink small amounts of water or other fluids throughout the day rather than trying to drink a large amount all at once. Sipping fluids slowly can help prevent symptoms such as nausea and bloating and can help ensure that your body is able to absorb the fluids properly.  If you are experiencing dehydration after weight loss surgery, it is important to discuss this with your surgeon or healthcare provider. They can recommend appropriate treatment options and can help you determine the cause of your dehydration. It is also important to follow their instructions for fluid intake after surgery to help ensure proper hydration and healing. | 3, 1 | 2, 1 | 3, 1 |
| Is it normal to get thrush after weight loss surgery? | Thrush is a fungal infection that can affect the mouth and throat. It is caused by an overgrowth of the Candida fungus, which is normally present in the mouth in small amounts. Thrush can cause symptoms such as white or yellow patches on the tongue and inside of the cheeks, redness, and soreness in the mouth and throat.  It is not uncommon to develop thrush after weight loss surgery, especially if you are taking certain medications, such as antibiotics, or if your immune system is compromised. Weight loss surgery can affect the digestive system and may alter the balance of bacteria in the body, which can increase the risk of thrush and other infections.  If you are experiencing symptoms of thrush after weight loss surgery, it is important to talk to your healthcare provider. They can diagnose the infection and recommend appropriate treatment options, such as antifungal medication.  It is also important to practice good oral hygiene, such as brushing your teeth regularly and using a mouthwash, to help prevent thrush and other infections. If you have any concerns about your oral health after weight loss surgery, it is best to ask your healthcare provider for guidance. | Thrush is a type of yeast infection that can affect the mouth, throat, and other areas of the body. It is not considered normal to develop thrush after weight loss surgery, but it is possible for people to develop thrush or other infections after any type of surgery.  There are several factors that can increase the risk of developing thrush or other infections after weight loss surgery, including:  Use of certain medications: Some medications, such as antibiotics, can disrupt the balance of bacteria in the body and increase the risk of thrush and other infections.  Poor oral hygiene: Poor oral hygiene, including not brushing and flossing regularly, can increase the risk of thrush and other infections.  Impaired immune system: Having a compromised immune system can increase the risk of thrush and other infections.  If you are experiencing symptoms of thrush after weight loss surgery, it is important to contact your surgeon or healthcare provider. They can diagnose the cause of your symptoms and recommend appropriate treatment. It is also important to follow their instructions for maintaining good oral hygiene after surgery to help prevent thrush and other infections. | 3, 1 | 1 | 3, 1 |
| Is it normal to experience depression after weight loss surgery? | It is not uncommon to experience changes in mood, including depression, after weight loss surgery. This can be due to a number of factors, including changes in hormone levels, alterations in brain chemistry, and the stress of undergoing a major surgery.  It is important to be aware of the potential for mood changes after weight loss surgery and to seek help if necessary. If you are experiencing symptoms of depression, such as persistent feelings of sadness or hopelessness, difficulty concentrating, changes in sleep or appetite, or lack of energy, it is important to talk to your healthcare provider. They can evaluate your symptoms and recommend appropriate treatment options, such as therapy or medication.  It is also important to remember that weight loss surgery is just one tool to help with weight loss. It is important to make lifestyle changes, such as following a healthy diet and getting regular exercise, to support your weight loss goals and overall mental health. If you have any concerns about your mental health after weight loss surgery, it is best to ask your healthcare provider for guidance. | Similar | 1 | 1 | N/A |
| Why do you get cold sweats after weight loss surgery? | Cold sweats can be a symptom of a number of different conditions and can be caused by a variety of factors. After weight loss surgery, cold sweats may be caused by changes in the body's metabolism or by certain medications.  It is not uncommon to experience changes in body temperature and sweating patterns after weight loss surgery. This can be due to changes in hormone levels and the body's metabolism, as well as the use of certain medications, such as pain relievers or anti-anxiety medications.  If you are experiencing cold sweats after weight loss surgery and are concerned, it is important to talk to your healthcare provider. They can evaluate your symptoms and determine the cause of your cold sweats. In some cases, treatment may be necessary to manage your symptoms and improve your overall health.  It is also important to remember that weight loss surgery is a major procedure and it is normal to experience some discomfort and side effects as you recover. If you have any concerns about your symptoms after weight loss surgery, it is best to ask your healthcare provider for guidance. | Similar | 1 | 1 | N/A |
| Will bariatric surgery cause new joint pain? | Bariatric surgery, also known as weight loss surgery, can potentially cause joint pain in some people. This is because losing a significant amount of weight can put added stress on the joints, which can lead to pain.  In addition, weight loss surgery can cause changes in hormone levels and the body's metabolism, which can affect the joints and cause pain. It is not uncommon to experience changes in joint pain after weight loss surgery, especially in the first few months after the procedure.  If you are experiencing joint pain after weight loss surgery, it is important to talk to your healthcare provider. They can evaluate your symptoms and suggest appropriate treatment options, such as medications or physical therapy, to help manage your pain.  It is also important to remember that weight loss surgery is a major procedure and it is normal to experience some discomfort and side effects as you recover. If you have any concerns about your joint pain after weight loss surgery, it is best to ask your healthcare provider for guidance. | Bariatric surgery can lead to significant weight loss, which can help reduce the burden on joints and may lead to improvement in joint pain. However, it is also possible that bariatric surgery may cause new joint pain or exacerbate existing joint pain in some people.  There are several factors that can contribute to joint pain after weight loss surgery, including:  Rapid weight loss: Rapid weight loss can lead to changes in the body's metabolism and hormone levels, which can cause joint pain.  Poor body alignment: Losing a significant amount of weight can alter the body's alignment and cause joint pain.  Nutrient deficiencies: Weight loss surgery can affect the body's ability to absorb certain nutrients, which can lead to deficiencies and contribute to joint pain.  If you are experiencing joint pain after weight loss surgery, it is important to discuss this with your surgeon or healthcare provider. They can determine the cause of your joint pain and recommend appropriate treatment options. It is also important to follow their instructions for caring for yourself after surgery and to seek medical attention if your joint pain is severe or if it interferes with your daily activities. | 2, 1 | 1 | 2, 1 |
| Is it normal to have severe abdominal cramps after weight loss surgery? | It is not uncommon to experience abdominal cramps or discomfort after weight loss surgery. This can be due to a number of factors, including changes in the digestive system, the use of pain medication, and the body's response to the surgery.  Most abdominal cramps after weight loss surgery are mild and resolve on their own within a few days to a week. However, if you are experiencing severe abdominal cramps or discomfort that persists beyond a week after surgery, it is important to talk to your healthcare provider. They can evaluate your symptoms and determine the cause of your abdominal cramps. In some cases, treatment may be necessary to manage your symptoms and improve your overall health.  It is also important to remember that weight loss surgery is a major procedure and it is normal to experience some discomfort and side effects as you recover. If you have any concerns about your abdominal cramps after weight loss surgery, it is best to ask your healthcare provider for guidance. | Similar | 1 | 1 | N/A |
| Are issues with hearing loss common after weight loss surgery? | Hearing loss is not a common side effect of weight loss surgery. However, some people may experience changes in their hearing after surgery due to changes in hormone levels or the use of certain medications.  If you are experiencing changes in your hearing after weight loss surgery and are concerned, it is important to talk to your healthcare provider. They can evaluate your hearing and suggest appropriate treatment options, if necessary.  It is also important to remember that weight loss surgery is a major procedure and it is normal to experience some discomfort and side effects as you recover. If you have any concerns about your hearing after weight loss surgery, it is best to ask your healthcare provider for guidance. | Similar | 1 | 1 | N/A |
| After gastric bypass surgery is it recommended to wear a waist trainer? | It is not generally recommended to wear a waist trainer after gastric bypass surgery. Waist trainers are garments that are worn around the waist in order to shape the waist and reduce the appearance of fat. They work by compressing the waist and can cause discomfort or even pain if worn for an extended period of time.  After gastric bypass surgery, it is important to follow the instructions of your surgeon and medical team to ensure a successful recovery. This may include wearing a compression garment to help with swelling, but a waist trainer is not typically part of the recommended post-surgery care plan. It is important to allow your body to heal and to avoid any activities that may put unnecessary strain on your incision or abdominal muscles. If you are interested in using a waist trainer or other body-shaping garment, it is best to consult with your doctor or a medical professional to determine if it is appropriate for your specific situation. | Similar | 1 | 1 | N/A |
| How long after weight loss surgery can dairy be added to the diet? | The timing of when dairy can be added back into the diet after weight loss surgery can vary depending on the type of surgery you had and your individual recovery. In general, it is usually recommended to wait until you have fully recovered from surgery before introducing dairy products into your diet. This is because dairy products can be difficult to digest, especially in the early stages after surgery when your digestive system is still healing.  After gastric bypass surgery, it is generally recommended to wait at least 6-8 weeks before introducing dairy products into your diet. This allows time for your incisions to heal and for your digestive system to adjust to the changes caused by the surgery. When you do start to add dairy back into your diet, it is important to do so gradually and to pay attention to your body's reaction. Some people may be able to tolerate small amounts of dairy without any problems, while others may experience digestive issues or discomfort after consuming dairy. If you experience any adverse reactions after eating dairy, it is a good idea to speak with your doctor or a medical professional for guidance. | Similar | 1 | 1 | N/A |
| Is it normal to have difficulty swallowing after weight loss surgery? | It is not uncommon to experience difficulty swallowing after weight loss surgery, especially in the early stages of recovery. Difficulty swallowing, also known as dysphagia, can be caused by a number of factors, including swelling in the throat, changes to the muscles and tissues in the throat and esophagus, or scar tissue from the surgery.  Dysphagia is typically more common after surgery that involves changes to the size or shape of the stomach, such as gastric bypass or sleeve gastrectomy. It can also be more common in people who have had previous surgery in the throat or esophagus.  If you are experiencing difficulty swallowing after weight loss surgery, it is important to speak with your doctor or a medical professional for guidance. They can evaluate your symptoms and determine the cause of the difficulty swallowing. In most cases, difficulty swallowing after weight loss surgery is temporary and will improve with time as your body adjusts to the changes caused by the surgery. However, it is important to address any issues with swallowing as soon as possible to ensure that you are able to eat and drink enough to meet your nutritional needs. | Similar | 1 | 1 | N/A |
| Why is acid reflux worse after weight loss surgery? | There are several reasons why acid reflux may be worse after weight loss surgery. Acid reflux, also known as gastroesophageal reflux disease (GERD), is a condition in which stomach acid flows back into the esophagus, causing a burning sensation in the chest and throat.  After weight loss surgery, it is not uncommon for people to experience an increase in acid reflux. This can be due to a number of factors, including:  Changes to the stomach: Weight loss surgery, such as gastric bypass or sleeve gastrectomy, can alter the size and shape of the stomach, which can affect the way that food and acid are processed.  Changes to the esophagus: Weight loss surgery can also affect the muscles and tissues of the esophagus, which can make it more prone to acid reflux.  Eating habits: After weight loss surgery, people often have to follow a specific diet that is designed to promote weight loss and healing. This may involve eating smaller, more frequent meals and avoiding certain types of food, such as those that are high in fat or acid. These changes to the diet can sometimes lead to an increase in acid reflux.  Lifestyle factors: Weight loss surgery can also lead to changes in lifestyle habits, such as increased physical activity or changes to sleep patterns. These changes can sometimes contribute to acid reflux.  If you are experiencing acid reflux after weight loss surgery, it is important to speak with your doctor or a medical professional for guidance. They can help to determine the cause of the acid reflux and recommend treatment options to help manage the condition. | Similar | 1 | 2 | 2 |
| Does weight loss surgery cause anemia? | Weight loss surgery can potentially cause anemia, although this is not a common complication. Anemia is a condition in which you have a deficiency of red blood cells or hemoglobin, which is the protein in red blood cells that carries oxygen to the body's tissues.  There are several ways in which weight loss surgery could potentially lead to anemia. For example, if you have a procedure such as gastric bypass surgery, your smaller stomach size may cause you to eat less and absorb fewer nutrients, including iron, which can lead to anemia. Additionally, some weight loss surgery procedures, such as gastric sleeve surgery, remove part of the stomach where iron is absorbed, which may also contribute to anemia.  It is important to monitor your nutrient levels, including your iron levels, after weight loss surgery and to follow the nutrition recommendations provided by your healthcare team. If you are at risk of developing anemia or if you already have anemia, your healthcare provider may recommend taking iron supplements or other medications to treat the condition. | Weight loss surgery can increase the risk of developing anemia, which is a condition that occurs when there is a deficiency of red blood cells or hemoglobin in the blood. Hemoglobin is a protein in red blood cells that carries oxygen to the body's tissues.  There are several factors that can contribute to the development of anemia after weight loss surgery. For example, the rapid weight loss that often occurs after bariatric surgery can cause the body to break down stored iron, leading to a deficiency. In addition, weight loss surgery may cause changes in the digestive system that make it more difficult to absorb nutrients, including iron, from the diet.  It's important to monitor your iron levels and to follow the recommendations of your healthcare team to prevent or treat anemia after weight loss surgery. This may include taking iron supplements, eating iron-rich foods, and getting regular blood tests to check your hemoglobin levels. If you are experiencing symptoms of anemia, such as fatigue, weakness, or shortness of breath, it's important to speak with your doctor to determine the cause and find a solution. | 3, 1 | 1 | 3, 1 |
| Why are there so many vitamins to take after weight loss surgery? | There are often a number of vitamins and minerals that are recommended after weight loss surgery to help ensure that you are getting sufficient nutrients. This is because weight loss surgery, such as gastric bypass surgery or gastric sleeve surgery, can significantly alter your body's ability to digest and absorb food. As a result, you may be at risk of nutrient deficiencies, especially in the early stages after surgery.  The specific vitamins and minerals that are recommended after weight loss surgery may vary depending on the type of surgery you had and your individual nutritional needs. Common vitamins and minerals that may be recommended include:  Vitamin B12: This vitamin is important for the production of red blood cells and the proper functioning of the nervous system. It is often recommended after weight loss surgery because it is absorbed in the small intestine, which may be bypassed during certain types of weight loss surgery.  Iron: This mineral is important for the production of red blood cells and the transport of oxygen in the body. It is often recommended after weight loss surgery because iron is absorbed in the stomach and upper small intestine, which may be removed during certain types of surgery.  Calcium and vitamin D: These nutrients are important for the maintenance of healthy bones. They may be recommended after weight loss surgery because calcium is absorbed in the small intestine, which may be bypassed during certain types of surgery, and because vitamin D is needed to absorb calcium.  It is important to follow the vitamin and mineral recommendations provided by your healthcare team after weight loss surgery to ensure that you are getting sufficient nutrients. | There are a number of reasons why people may be advised to take vitamins after weight loss surgery. One of the main reasons is that weight loss surgery, such as gastric bypass or gastric sleeve surgery, can significantly alter the body's ability to absorb nutrients from food. This can lead to deficiencies in essential vitamins and minerals that are important for maintaining good health.  For example, after weight loss surgery, people may be at risk of developing deficiencies in vitamins such as B12, iron, and folate, as well as minerals such as calcium and zinc. Taking a daily multivitamin and other supplements can help to ensure that you are getting enough of these essential nutrients to support your health.  It's important to follow the recommendations of your healthcare team when it comes to taking vitamins after weight loss surgery. Your doctor or a registered dietitian can help you determine which vitamins and supplements are appropriate for you based on your individual needs and any deficiencies that may exist. | 3, 1 | 1 | 3, 1 |
| How long after weight loss surgery can you exercise? | The length of time it takes to recover from weight loss surgery and when you can start exercising again will vary depending on the type of surgery you had and your individual recovery.  In general, it is important to follow the exercise guidelines provided by your surgeon and to listen to your body. You may need to take it easy for the first few weeks after surgery and gradually increase your activity as you heal. This may include starting with gentle activities such as walking and gradually increasing the intensity and duration of your workouts as tolerated.  It is important to consult with your surgeon or a healthcare professional before starting an exercise program after weight loss surgery. They will be able to provide you with specific recommendations based on your individual situation and needs.  It is also important to note that weight loss surgery is just one tool to help with weight loss and maintenance, and regular physical activity is an essential component of a healthy lifestyle. Aim to get at least 150 minutes of moderate-intensity aerobic activity (such as brisk walking) or 75 minutes of vigorous-intensity aerobic activity (such as jogging) per week, as well as muscle-strengthening activities at least two days per week. | Similar | 1 | 1 | N/A |
| Will weight loss surgery make you more likely to fall sick? | Weight loss surgery is generally not associated with an increased risk of falling sick. In fact, weight loss surgery can often improve overall health and reduce the risk of certain medical conditions, such as type 2 diabetes, high blood pressure, and heart disease.  However, as with any major surgery, there are potential risks and complications associated with weight loss surgery. It is important to discuss the potential risks and benefits of weight loss surgery with a healthcare professional and to follow the post-surgery care instructions provided by your surgeon to help minimize the risk of complications.  It is also important to note that weight loss surgery is just one tool to help with weight loss and maintenance, and a healthy lifestyle including a balanced diet and regular physical activity is essential for long-term success. | Weight loss surgery itself is not likely to make you more prone to illness. In fact, weight loss surgery can have a number of health benefits, including improving or resolving conditions such as diabetes, high blood pressure, and sleep apnea.  However, it's important to be aware that the rapid weight loss that often occurs after weight loss surgery can sometimes weaken the immune system, making you more susceptible to infections. This is because the body needs time to adjust to the changes that have occurred after surgery, and losing a large amount of weight in a short period of time can put additional stress on the body.  To help minimize the risk of infection and other complications after weight loss surgery, it's important to follow the recommendations of your healthcare team, including getting vaccinated, practicing good hygiene, and taking any prescribed medications as directed. It's also a good idea to eat a healthy diet and get enough rest to help support your immune system and overall health. | 2, 1 | 1 | 2, 1 |
| How long after weight loss surgery can you be sexually active? | The length of time it takes to recover from weight loss surgery and when you can resume sexual activity will vary depending on the type of surgery you had and your individual recovery.  It is important to follow the post-surgery instructions provided by your surgeon and to listen to your body. You may need to take it easy for the first few weeks after surgery and gradually increase your activity as you heal. This may include avoiding strenuous activities, such as sexual activity, until you are fully recovered.  It is also important to remember that every person is different and may recover at a different rate. It is a good idea to discuss your specific situation with your surgeon or a healthcare professional to get a better idea of when it is safe to resume sexual activity after weight loss surgery.  It is also important to use caution and proper protection during sexual activity to reduce the risk of infection or other complications. | Similar | 1 | 1 | N/A |
| Does weight loss surgery increase risk of hernias? | Weight loss surgery may increase the risk of hernias. A hernia is a condition in which an organ or tissue pushes through an opening in the muscle or tissue that holds it in place. There are several types of hernias, including abdominal hernias, which can occur after weight loss surgery.  Weight loss surgery, such as gastric bypass surgery or gastric sleeve surgery, involves making incisions in the abdominal wall, which can weaken the muscles and increase the risk of hernias. In addition, the rapid weight loss that often occurs after weight loss surgery can also put additional strain on the abdominal muscles and increase the risk of hernias.  It is important to follow the post-surgery instructions provided by your surgeon and to report any unusual symptoms, such as pain or swelling, to a healthcare professional. If a hernia is suspected, your healthcare provider will be able to recommend the appropriate treatment. | Weight loss surgery, also known as bariatric surgery, can increase the risk of developing hernias. A hernia occurs when an organ or tissue protrudes through a weak point in the muscles of the abdominal wall. There are several types of weight loss surgery, including gastric bypass, laparoscopic adjustable gastric band, and sleeve gastrectomy, and each procedure carries a different risk of hernia.  The risk of developing a hernia after weight loss surgery is higher in people who are obese, have a history of hernias, or have other risk factors such as tobacco use or heavy lifting. In general, the risk of hernia after weight loss surgery is relatively low, but it is important for patients to be aware of the potential for hernias and to seek medical attention if they experience any symptoms. Symptoms of a hernia may include a bulge in the abdominal area, pain or discomfort, and difficulty with bowel movements. | 1 | 2 | 1, 2 |
| Is it normal to have chest pain months after weight loss surgery? | It is generally not normal to have chest pain after weight loss surgery, especially if it is occurring months after the procedure. Chest pain can be a sign of a serious medical condition, such as a heart attack, and should not be ignored.  If you are experiencing chest pain after weight loss surgery, it is important to contact a healthcare professional immediately. They will be able to assess your symptoms and determine the cause of the chest pain.  It is also a good idea to keep track of your symptoms, including the location, intensity, and duration of the chest pain, and to bring this information with you to your appointment. This will help your healthcare provider make a diagnosis and determine the appropriate treatment. | Similar | 1 | 1 | N/A |
| How long does fatigue after weight loss surgery last? | The length of time fatigue lasts after weight loss surgery will vary depending on the individual and the specific type of surgery they had. Some people may experience fatigue for a few weeks after surgery, while others may continue to feel tired for several months or longer.  Fatigue after weight loss surgery can be caused by a number of factors, including the surgery itself, changes in diet and activity level, and the body's response to the changes brought about by the surgery. It is also common for people to experience fatigue as they adjust to the physical and emotional demands of the weight loss journey.  If you are experiencing fatigue after weight loss surgery, it is important to listen to your body and give yourself time to rest and recover. It is also a good idea to speak with a healthcare professional if your fatigue persists or is affecting your daily activities. They can help determine the cause of your fatigue and recommend strategies to manage it. | Similar | 1 | 1 | N/A |
| Is excessive burping normal after weight loss surgery? | Excessive burping can be a common side effect after weight loss surgery, particularly after gastric bypass surgery or sleeve gastrectomy. This can be due to the altered anatomy of the stomach and the small size of the pouch, which can cause food to pass through the stomach more quickly and may result in increased gas production.  If you are experiencing excessive burping after weight loss surgery, it is a good idea to speak with a healthcare professional. They will be able to assess your symptoms and determine the cause of the burping. In some cases, making changes to your diet or taking over-the-counter remedies may be helpful in reducing burping.  It is also important to follow the post-surgery instructions provided by your surgeon and to report any unusual symptoms to a healthcare professional. They will be able to provide guidance and recommend treatment if necessary. | Similar | 1 | 1 | N/A |
| When do the diet restrictions end after weight loss surgery? | The diet restrictions after weight loss surgery will vary depending on the type of surgery you had and your individual needs and goals. In general, the diet restrictions after weight loss surgery are intended to help you adjust to your new anatomy and to promote weight loss.  After weight loss surgery, you will typically start with a liquid diet for the first few weeks, followed by a progression to soft foods and then to solid foods. The specific foods you are allowed to eat and the portion sizes will depend on the type of surgery you had and your individual needs.  As you progress through the post-surgery diet stages, you will typically be able to gradually return to a more normal diet. However, it is important to continue to follow the dietary recommendations provided by your healthcare team to ensure that you are getting sufficient nutrients and to help prevent weight regain.  It is also important to remember that weight loss surgery is just one tool to help with weight loss and maintenance, and a healthy lifestyle including a balanced diet and regular physical activity is essential for long-term success. | Similar | 1 | 1 | N/A |
| What causes foaming at the mouth after weight loss surgery? | Foaming at the mouth after weight loss surgery may be caused by a variety of factors, including:  Excess air swallowing: After weight loss surgery, it is common for people to swallow more air due to changes in the digestive system and the small size of the pouch. This can lead to bloating and excess gas, which may cause foaming at the mouth.  Infection: Foaming at the mouth may be a sign of an infection, such as pneumonia, in the mouth or respiratory system.  Allergic reaction: Foaming at the mouth may occur in some individuals as a symptom of an allergic reaction.  Medication side effect: Some medications, such as certain antipsychotic medications, can cause foaming at the mouth as a side effect.  If you are experiencing foaming at the mouth after weight loss surgery, it is important to speak with a healthcare professional as soon as possible. They will be able to assess your symptoms and determine the cause of the foaming. | Similar | 1 | 1 | N/A |
| How long after weight loss surgery can marijuana edibles be consumed? | It is generally not recommended to consume marijuana edibles after weight loss surgery, as the effects of marijuana on the body can interfere with the healing process and may negatively impact weight loss outcomes.  After weight loss surgery, it is important to follow the dietary and activity recommendations provided by your healthcare team to promote healing and optimize weight loss. This may include avoiding certain foods and substances, including marijuana, that could interfere with the healing process or contribute to weight gain.  It is also important to note that marijuana is a psychoactive substance and can have potentially harmful effects on the body, even when consumed in edible form. The use of marijuana can impair judgment, coordination, and memory, and may have negative effects on mental health.  If you are considering using marijuana after weight loss surgery, it is a good idea to speak with a healthcare professional to determine if it is a safe and appropriate option for you. They can provide guidance on the potential risks and benefits and help you make an informed decision about your use of marijuana. | It is generally recommended to wait at least several weeks after weight loss surgery before consuming marijuana edibles. This is because the surgery can significantly alter the way the body processes and metabolizes medications and substances, including marijuana. In the immediate period after surgery, it is important to follow a specific diet and medication regimen that has been recommended by your healthcare team. Consuming marijuana edibles during this time could interfere with your recovery and the success of the surgery. It is important to discuss your use of marijuana and any other substances with your healthcare team before and after surgery to ensure the safety of your treatment and recovery. | 1, 3 | 1 | 1, 3 |
| Is ankle swelling normal after weight loss surgery? | It is not uncommon for people to experience swelling in their ankles after weight loss surgery. This is often due to a condition called edema, which is the accumulation of fluid in the body's tissues. The swelling can be caused by a variety of factors, including changes in diet and activity level, changes in hormone levels, and the body's response to surgery. In most cases, ankle swelling after weight loss surgery is not a cause for concern and can be managed with simple self-care measures, such as elevating the legs, wearing compression stockings, and staying hydrated. If the swelling is severe or persists for an extended period of time, it is important to speak with a healthcare provider for further evaluation and treatment. | Ankle swelling can occur after weight loss surgery, although it is not necessarily a normal or expected side effect. Swelling in the ankles, also known as edema, can be caused by a number of factors, including excess fluid accumulation, inflammation, or poor circulation. If you are experiencing ankle swelling after weight loss surgery, it is important to speak with your healthcare provider as soon as possible, as it could be a sign of a more serious underlying issue that needs to be addressed. Possible causes of ankle swelling after weight loss surgery could include a blood clot, an infection, or a dietary deficiency, among other things. Your healthcare provider will be able to evaluate your symptoms and recommend appropriate treatment. | 2, 2 | 1 | 2, 2 |
| Is ketosis normal after weight loss surgery? | It is not uncommon for people to experience ketosis after weight loss surgery, especially in the early postoperative period. Ketosis is a metabolic state in which the body burns fat for fuel instead of carbohydrates. It is characterized by the presence of ketones in the blood and urine.  There are several reasons why ketosis may occur after weight loss surgery. One reason is that the surgery may alter the body's ability to absorb nutrients, leading to a deficiency in carbohydrates. This can cause the body to switch to burning fat for fuel. In addition, the rapid weight loss that often occurs after surgery may also contribute to ketosis.  While ketosis is generally not harmful, it is important to monitor your ketone levels and make sure that they do not become too high. If you are experiencing ketosis after weight loss surgery, it is important to speak with your healthcare provider for guidance on how to manage it. They may recommend adjusting your diet or taking other measures to help regulate your ketone levels. | It is not necessarily normal or expected to experience ketosis after weight loss surgery. Ketosis is a metabolic state in which the body begins to burn fat for fuel instead of carbohydrates. It can occur when the body does not have sufficient carbohydrates available to use for energy, and it is often a goal of certain types of low-carb diets.  After weight loss surgery, it is common for people to experience significant weight loss and to need to make changes to their diet in order to support their new weight and ensure they are getting adequate nutrition. However, it is generally not recommended to intentionally try to induce ketosis after weight loss surgery, as it can be difficult to get enough nutrients while following a very low-carb diet.  If you are experiencing ketosis after weight loss surgery, it is important to speak with your healthcare provider or a registered dietitian to ensure that you are getting adequate nutrition and to address any potential underlying issues. They will be able to provide guidance on appropriate nutrition and make any necessary recommendations. | 3, 1 | 1 | 3, 1 |
| How long after weight loss surgery can you use a straw? | The use of straws after weight loss surgery may depend on the type of procedure you had and the stage of your recovery. In general, it is important to follow the guidelines provided by your healthcare team and to avoid activities that could cause strain or discomfort.  After certain types of weight loss surgery, such as gastric bypass or gastric sleeve surgery, the size of the stomach is significantly reduced. This can make it more difficult to eat and drink enough to meet your nutritional needs. To help increase your intake of fluids and calories, your healthcare provider may recommend that you use a straw to drink liquids, particularly in the early postoperative period. Using a straw can help to bypass the smaller stomach and deliver fluids directly to the intestines, where they can be absorbed more easily.  However, it is important to be mindful of the type and amount of fluids you consume using a straw. Some liquids, such as carbonated beverages or high-calorie drinks, may not be appropriate or may interfere with your weight loss goals. In addition, using a straw excessively or too soon after surgery can cause discomfort or cause the stomach to stretch, which may lead to discomfort or complications. If you are considering using a straw after weight loss surgery, it is important to speak with your healthcare provider or a dietitian for guidance on how to do so safely and effectively. | It is generally recommended to avoid using a straw for the first few weeks after weight loss surgery, as sucking on a straw can put added strain on the surgical site and may cause discomfort. After this initial period, it may be okay to use a straw, although it is important to do so with caution and to listen to your body.  If you experience discomfort or difficulty while using a straw, it is important to stop and speak with your healthcare provider or a registered dietitian. They will be able to provide guidance on when it is appropriate to resume using a straw and can offer advice on how to do so safely.  It is also important to keep in mind that it is generally recommended to take small, frequent sips of fluids rather than large amounts at once after weight loss surgery. This can help to reduce the risk of discomfort and can also help to prevent dehydration. It is a good idea to talk to your healthcare provider or a registered dietitian for specific recommendations on how much and how often to drink fluids after weight loss surgery. | 4, 1 | 4, 3 | 4, 4 |
| What does a typical diet right after weight loss surgery include? | The diet after weight loss surgery will depend on the type of procedure you had and the stage of your recovery. In general, it is important to follow the guidelines provided by your healthcare team and to focus on consuming a balanced, nutrient-dense diet that meets your nutritional needs.  In the early postoperative period, it is common for people to follow a liquid or pureed diet to help the stomach heal and to allow the body to adjust to the changes made during surgery. This may include foods such as broth, protein shakes, pureed soups, and pureed meats. As you progress through the postoperative period, you may be able to gradually add more solid foods back into your diet.  It is important to consume adequate protein to help support healing and maintain muscle mass. You may also need to take supplements, such as a multivitamin, to ensure that you are getting enough nutrients. Your healthcare team will provide specific guidelines on what to eat and drink after surgery, including portion sizes and the frequency of meals. They will also be available to answer any questions you may have and to provide support and guidance as you adjust to your new diet. | Similar | 1 | 1 | N/A |
| Are fatty stools normal after weight loss surgery? | Fatty stools, also known as steatorrhea, may occur after weight loss surgery, particularly after procedures that involve the removal or bypassing of a portion of the small intestine, such as gastric bypass or duodenal switch surgery. When a portion of the small intestine is removed or bypassed, it can lead to malabsorption of fat and other nutrients, which can cause fatty stools.  Fatty stools may be characterized by an oily or greasy appearance, a foul odor, and a floating or difficult-to-flush consistency. They may also be accompanied by other symptoms, such as abdominal pain, bloating, and diarrhea. If you are experiencing fatty stools after weight loss surgery, it is important to speak with your healthcare provider for further evaluation and treatment. They may recommend dietary changes, supplements, or other interventions to help manage the problem and ensure that you are getting adequate nutrition. | Similar | 1 | 1 | N/A |
| Does weight loss surgery increase risk for blood clots? | Weight loss surgery, or bariatric surgery, may increase the risk of developing blood clots, particularly in the first few weeks after surgery. This is because surgery, particularly when combined with immobility, can increase the risk of blood clots forming in the legs, a condition known as deep vein thrombosis (DVT).  However, it is important to note that the risk of developing blood clots after weight loss surgery can be managed and minimized through a combination of preventive measures, including medication, compression stockings, and early ambulation (moving around).  If you are scheduled to have weight loss surgery and have concerns about the risk of blood clots, it is important to speak with your healthcare provider. They will be able to provide specific recommendations based on your individual circumstances and medical history, and can help to develop a plan to minimize your risk of developing blood clots after surgery. | Similar | 1 | 1 | N/A |
| **Postoperative Lifestyle Changes (4)** | | | | | |
| Is it okay to take antacids like Tums after weight loss surgery? | Antacids, such as Tums, are medications that are used to neutralize stomach acid and relieve symptoms of heartburn, indigestion, and acid reflux. It is generally safe to take antacids after weight loss surgery, but it is important to follow your healthcare provider's recommendations and to use them as directed.  After weight loss surgery, the digestive system may be slower and less efficient, which can lead to symptoms such as heartburn and indigestion. Antacids can be helpful in relieving these symptoms, but it is important to use them sparingly and as needed, rather than relying on them regularly.  If you are experiencing persistent or severe symptoms of heartburn or indigestion after weight loss surgery, it is important to talk to your healthcare provider. They can evaluate your symptoms and suggest appropriate treatment options, such as changes to your diet or the use of other medications.  If you have any concerns about using antacids after weight loss surgery, it is best to ask your healthcare provider for guidance. | Similar | 1 | 1 | N/A |
| Will you have to exercise after the weight loss surgery? | Exercise is an important part of the weight loss journey, especially after weight loss surgery. Engaging in regular physical activity can help you lose weight and keep it off, improve your cardiovascular health, and boost your mood and energy levels. However, the specific exercise recommendations after weight loss surgery may vary depending on the type of surgery you have and your individual circumstances. It is important to follow the exercise guidelines provided by your healthcare team and to start slowly, gradually increasing the intensity and duration of your workouts as you become more comfortable. It is also important to listen to your body and avoid overexerting yourself. | Exercise is an important part of maintaining a healthy weight and lifestyle after weight loss surgery. Regular physical activity can help you lose weight and keep it off, improve your overall health, and reduce the risk of certain medical conditions, such as heart disease, diabetes, and high blood pressure. However, the specific exercise recommendations after weight loss surgery will depend on the type of surgery you had and your individual needs and goals. In general, it is recommended to aim for at least 150 minutes of moderate-intensity aerobic exercise, such as brisk walking or cycling, per week, as well as strength training at least two days per week. It is also important to incorporate flexibility and balance exercises into your routine. It is always a good idea to consult with your surgeon or healthcare provider before starting any new exercise program to make sure it is safe and appropriate for you. | 2 | 2, 1 | 2, 1 |
| Will you have to be on a diet after the weight loss surgery? | After weight loss surgery, it is important to follow a healthy diet to help you lose weight and support your overall health. The specific dietary recommendations after weight loss surgery may vary depending on the type of surgery you have and your individual circumstances. However, in general, it is important to: - Eat a balanced and nutrient-dense diet that includes a variety of fruits, vegetables, lean proteins, and whole grains. - Drink plenty of fluids, including water, to stay hydrated and help your body function properly. - Avoid sugary drinks and foods that are high in added sugars and unhealthy fats. - Eat slowly and chew your food well to help your body digest food properly and prevent digestive issues. - Pay attention to your body's hunger and fullness cues and stop eating when you are satisfied, rather than stuffed. It is also important to follow the dietary guidelines provided by your healthcare team and to make any necessary adjustments as you progress through the recovery process. They can provide guidance and support to help you make healthy dietary choices and achieve your weight loss goals. | Similar | 2 | 1 | 2 |
| Will shoe size change or decrease after weight loss surgery? | It is possible that shoe size may change after weight loss surgery, as weight loss can affect the shape and size of the feet. Rapid weight loss, in particular, may cause the feet to become smaller and narrower.  If you experience a significant change in shoe size after weight loss surgery, it is generally recommended to purchase new shoes to ensure a proper fit. Ill-fitting shoes can cause discomfort and may increase the risk of foot problems, such as blisters or calluses.  It is generally recommended to follow the guidelines provided by your healthcare team for diet and lifestyle after weight loss surgery to help maintain your weight loss and promote a healthy recovery. This may include eating a balanced diet that is rich in protein, fiber, and nutrients, getting regular physical activity, and managing stress. Your healthcare team can provide you with more specific recommendations based on your individual needs and the type of weight loss surgery you have undergone. | Similar | 1 | 1 | N/A |
| Can you take electrolytes after weight loss surgery? | It is generally recommended to take electrolytes after weight loss surgery to help maintain proper fluid and electrolyte balance in the body. Electrolytes are minerals, such as sodium, potassium, and magnesium, that are essential for proper body function. They help to regulate the balance of fluids in the body and play a critical role in many important bodily processes, including muscle function, heart function, and nerve function.  After weight loss surgery, it is important to pay close attention to your electrolyte levels, as the surgery can affect the body's ability to absorb and process electrolytes. If you are not getting enough electrolytes, you may experience symptoms such as fatigue, muscle weakness, or irregular heartbeat.  It is generally recommended to follow the guidelines provided by your healthcare team for diet and nutrition after weight loss surgery to help ensure that you are getting enough electrolytes. This may include taking electrolyte supplements as prescribed, eating foods that are rich in electrolytes, or drinking electrolyte-rich fluids. Your healthcare team can provide you with more specific recommendations based on your individual needs and the type of weight loss surgery you have undergone. | Similar | 1 | 1 | N/A |
| Is smoking after weight loss surgery allowed? | Smoking is generally not recommended after weight loss surgery, as it can increase the risk of complications and undermine the benefits of the procedure.  Smoking can impair the healing process and increase the risk of infections, wound complications, and other problems after weight loss surgery. In addition, smoking can interfere with the body's metabolism and make it more difficult to maintain weight loss after the procedure.  If you are a smoker and are considering weight loss surgery, it is important to discuss your smoking habits with your healthcare team and to make a plan to quit smoking before and after the surgery. Your healthcare team can provide you with resources and support to help you quit smoking and can help you to understand the potential risks and benefits of weight loss surgery in the context of your smoking habits.  It is generally recommended to follow the guidelines provided by your healthcare team for diet and lifestyle after weight loss surgery to help promote a healthy recovery and maximize the benefits of the procedure. This may include avoiding smoking and other unhealthy behaviors, eating a balanced diet, getting regular physical activity, and managing stress. | It is generally not recommended that people smoke after weight loss surgery. Smoking can have a number of negative effects on the body and can interfere with the healing process after surgery. In addition, smoking can increase the risk of complications such as infection, bleeding, and respiratory problems after weight loss surgery.  If you are a smoker and are considering weight loss surgery, it is important to discuss your smoking habit with your surgeon or healthcare provider. They may recommend that you quit smoking before surgery or may provide resources to help you quit smoking. It is also important to follow the instructions of your healthcare team regarding smoking after weight loss surgery to help ensure a successful outcome and to reduce the risk of complications. | 1 | 2 | 1, 2 |
| How do you prevent weight gain years after weight loss surgery? | Weight regain after weight loss surgery is a common concern. Here are some tips that may help you prevent weight gain after weight loss surgery:  Follow a healthy diet: It's important to eat a well-balanced diet that includes a variety of nutrients, including protein, fiber, and healthy fats. It's also important to focus on portion sizes and avoid overeating.  Exercise regularly: Regular physical activity can help you maintain your weight loss and improve your overall health. Aim for at least 150 minutes of moderate-intensity exercise per week.  Seek support: Consider joining a support group or working with a registered dietitian or other healthcare professional to help you stay on track with your weight loss goals.  Monitor your weight: Regularly monitoring your weight can help you catch any small gains before they become bigger problems.  Seek medical attention if necessary: If you are struggling to maintain your weight loss, it may be helpful to talk to your healthcare team about possible medical causes of weight regain and ways to address them.  It's also important to remember that weight loss surgery is just one tool to help with weight loss. It's important to make lifestyle changes, such as following a healthy diet and getting regular exercise, to support your weight loss goals in the long term. | Similar | 1 | 1 | N/A |
| Why do you have to drink 64 ounces of water each day after weight loss surgery? | Drinking 64 ounces (about 2 liters) of water per day after weight loss surgery is generally recommended to help prevent dehydration and to support the healing process. Weight loss surgery, such as gastric bypass or sleeve gastrectomy, can cause changes to the digestive system that can affect the way that the body absorbs fluids. This can increase the risk of dehydration, especially in the early stages after surgery when the body is still adjusting to the changes.  In addition to helping to prevent dehydration, drinking 64 ounces of water per day after weight loss surgery can also help to flush out toxins and waste products from the body, support the healing process, and improve digestion. Water is an essential nutrient that is necessary for many of the body's functions, and it is important to get enough of it to stay hydrated and healthy.  It is generally recommended to drink 64 ounces of water per day after weight loss surgery, but the specific amount of water that you need may vary depending on your age, gender, size, and activity level. It is important to follow the instructions of your surgeon and medical team and to pay attention to your body's thirst cues to ensure that you are getting enough water. If you are having trouble meeting this goal, you can try drinking water throughout the day or adding water-rich foods, such as fruits and vegetables, to your diet. | Similar | 1 | 1 | N/A |
| Is it okay to follow the keto diet after weight loss surgery? | It is generally not recommended to follow the ketogenic diet (also known as the keto diet) after weight loss surgery. The keto diet is a high-fat, low-carbohydrate diet that is designed to induce ketosis, a metabolic state in which the body burns fat for fuel instead of carbohydrates. While the keto diet may be effective for weight loss in some people, it may not be appropriate for everyone, especially after weight loss surgery.  After weight loss surgery, it is important to follow a balanced diet that provides all of the nutrients that your body needs to heal and to support long-term health. The keto diet is very low in carbohydrates, which can be an important source of energy and nutrients, including fiber, vitamins, and minerals. Cutting out or severely limiting carbohydrates can make it difficult to get enough of these important nutrients, and it can also lead to other health problems, such as constipation and nutrient deficiencies.  If you are considering following the keto diet after weight loss surgery, it is important to speak with your doctor or a medical professional for guidance. They can help you to understand the potential risks and benefits of the diet and to determine if it is appropriate for you in your specific situation. They can also help you to develop a healthy, balanced diet plan that meets your nutritional needs and supports your weight loss goals. | It is generally safe to follow a ketogenic diet after weight loss surgery, but it's important to discuss this with your doctor or a registered dietitian first. Weight loss surgery, such as gastric bypass or gastric sleeve surgery, can significantly alter your body's ability to absorb nutrients and may require you to follow a specific diet in the months or years following the procedure.  A ketogenic diet can be high in fat and protein and low in carbohydrates, which can be difficult to follow after weight loss surgery due to the limited amount of food that can be consumed at one time. It's also important to ensure that you are getting enough nutrients, including protein, vitamins, and minerals, to support your body's needs.  In general, it's a good idea to work with a registered dietitian or a healthcare professional who is knowledgeable about nutrition and weight loss surgery to develop a safe and effective diet plan that meets your individual needs. | 1, 2 | 1, 2 | N/A |
| Will drinking alcohol after weight loss surgery cause weight regain? | Drinking alcohol after weight loss surgery can potentially contribute to weight regain if it is consumed in excess. Alcohol is high in calories and can interfere with your body's ability to properly metabolize nutrients. Additionally, alcohol can lower your inhibitions and lead to overeating or making poor food choices.  However, it is important to note that moderate alcohol consumption (defined as up to one drink per day for women and up to two drinks per day for men) is generally considered safe after weight loss surgery. It is also important to remember that weight loss surgery is just one tool to help with weight loss and maintenance, and a healthy lifestyle including a balanced diet and regular physical activity is essential for long-term success.  It is always a good idea to speak with your healthcare provider or surgeon about your alcohol consumption after weight loss surgery to ensure it is safe and appropriate for your specific situation. | Similar | 1 | 1 | N/A |
| What is the best diet to follow after weight loss surgery to prevent weight regain? | The best diet to follow after weight loss surgery to prevent weight regain will depend on your individual needs and goals. However, there are some general guidelines that can help you maintain your weight loss after surgery:  Eat a balanced diet that includes a variety of nutrient-dense foods, such as fruits, vegetables, lean proteins, and whole grains.  Drink plenty of water to stay hydrated and to help your body function properly.  Limit your intake of sugary and high-fat foods, as these can contribute to weight gain.  Eat small, frequent meals throughout the day to help manage hunger and prevent overeating.  Monitor your portion sizes and pay attention to your body's hunger and fullness cues.  Engage in regular physical activity to help maintain your weight loss and improve overall health.  It is also important to work with a registered dietitian or a healthcare professional to develop a personalized meal plan that meets your specific needs and goals after weight loss surgery. They can help you create a plan that is safe, nutritionally balanced, and sustainable in the long term. | Similar | 1 | 1 | N/A |
| How long do you take multivitamins for after weight loss surgery? | The length of time you should take multivitamins after weight loss surgery will depend on your individual needs and the specific recommendations of your healthcare team.  After weight loss surgery, it is common to have nutrient deficiencies due to the altered anatomy and reduced food intake. As a result, many people are advised to take multivitamins and other supplements to help ensure that they are getting sufficient nutrients.  The specific vitamins and minerals that you may need to take after weight loss surgery will depend on the type of surgery you had and your individual nutritional needs. Your healthcare team will be able to provide you with specific recommendations based on your situation.  It is important to follow the vitamin and mineral recommendations provided by your healthcare team after weight loss surgery to ensure that you are getting sufficient nutrients. It is also a good idea to speak with a healthcare professional or a registered dietitian if you have questions or concerns about your vitamin and mineral intake after weight loss surgery. | Similar | 1 | 1 | N/A |
| Is it recommended to start personal training months after weight loss surgery? | It is generally safe to start personal training months after weight loss surgery, as long as you have received clearance from a healthcare professional and are feeling physically ready. Personal training can be a helpful way to improve your fitness level, build strength, and achieve your weight loss and other health goals after surgery.  After weight loss surgery, it is common to have some restrictions on physical activity in the immediate post-surgery period. These restrictions are typically lifted gradually over time, and it is important to follow the guidance of your healthcare team to ensure that you are progressing safely.  If you are considering personal training after weight loss surgery, it is a good idea to speak with a healthcare professional to determine if it is a safe and appropriate option for you. They can provide guidance on the type and intensity of physical activity that is safe for you at this stage and can help you develop a safe and effective exercise plan.  It is also important to remember that personal training is just one component of a healthy lifestyle and that a balanced diet and regular physical activity are essential for long-term success. | Similar | 1 | 1 | N/A |
| Does fiber have to be restricted in diet after weight loss surgery? | The amount of fiber in the diet after weight loss surgery will depend on the type of surgery you had and your individual needs and goals.  After weight loss surgery, it is common to have some restrictions on fiber intake in the immediate post-surgery period to allow the digestive system to heal. As you progress through the post-surgery diet stages, you will typically be able to gradually increase your fiber intake.  However, it is important to follow the dietary recommendations provided by your healthcare team after weight loss surgery to ensure that you are getting sufficient nutrients and to help prevent weight regain. This may include limiting your intake of high-fiber foods, such as raw fruits and vegetables, until you are able to tolerate them.  It is also important to remember that fiber is an important nutrient that can help support digestive health and may have other health benefits. Getting sufficient fiber in the diet after weight loss surgery is important, and it is a good idea to speak with a healthcare professional or a registered dietitian if you have questions or concerns about your fiber intake. They can provide guidance on the appropriate amount of fiber for your individual needs. | Similar | 1 | 1 | N/A |
| Is it safe to use Tylenol after weight loss surgery? | It is generally safe to use Tylenol (acetaminophen) after weight loss surgery, as long as it is taken according to the recommended dosage. Tylenol is a widely used over-the-counter pain medication that is effective at relieving mild to moderate pain. It works by reducing the production of prostaglandins, chemicals that are involved in inflammation and pain.  After weight loss surgery, Tylenol may be used to help manage postoperative pain. However, it is important to follow the dosing instructions on the label or as directed by your healthcare provider. Taking too much Tylenol can cause serious side effects, including liver damage. It is also important to avoid taking Tylenol if you have certain medical conditions or are taking certain medications, as it may not be safe for you. If you have any questions or concerns about using Tylenol after weight loss surgery, it is important to speak with a healthcare provider for guidance. | Similar | 1 | 1 | N/A |
| Is it okay to chew gum after weight loss surgery? | Chewing gum may be okay after weight loss surgery, depending on the type of procedure you had and the stage of your recovery. In general, it is important to follow the guidelines provided by your healthcare team and to avoid activities that could cause strain or discomfort.  After certain types of weight loss surgery, such as gastric bypass or gastric sleeve surgery, the size of the stomach is significantly reduced. This can make it more difficult to eat and drink enough to meet your nutritional needs. To help increase your intake of fluids and calories, your healthcare provider may recommend that you chew gum to stimulate saliva production and to keep your mouth moist. Chewing gum may also help to reduce hunger and cravings for sweet or high-calorie foods.  However, it is important to be mindful of the type and amount of gum you chew. Some types of gum contain sugar or other sweeteners that can contribute to tooth decay or interfere with weight loss goals. In addition, chewing gum excessively or too soon after surgery can cause discomfort or cause the stomach to stretch, which may lead to discomfort or complications. If you are considering chewing gum after weight loss surgery, it is important to speak with your healthcare provider or a dietitian for guidance on how to do so safely and effectively. | Chewing gum may be okay to consume in moderation after weight loss surgery, depending on the type of surgery you have had and your individual circumstances. It is generally recommended to avoid chewing gum in the first few weeks after surgery, as the act of chewing can put added strain on the surgical site and may cause discomfort.  After this initial period, chewing gum may be okay as long as it is sugar-free and does not cause any discomfort or digestive issues. Some people may find that chewing gum can help to reduce feelings of hunger or promote saliva production, which can be beneficial after weight loss surgery. However, it is important to keep in mind that gum is not a substitute for proper nutrition and should not be relied on as a primary source of calories or nutrients.  It is always a good idea to talk to your healthcare provider or a registered dietitian for specific recommendations on what to eat and drink after weight loss surgery. They will be able to provide guidance based on your individual needs and circumstances. | 1 | 1, 2 | 3,3 |
| How do you build back muscle mass after weight loss surgery? | There are several steps you can take to build back muscle mass after weight loss surgery:  Consult with a healthcare provider or physical therapist to determine a safe and appropriate exercise plan.  Gradually increase your activity level. Start with low-impact exercises, such as walking or swimming, and gradually progress to more intense activities as your strength and endurance improve.  Focus on strength training exercises that target all major muscle groups, including the chest, arms, shoulders, back, legs, and core. This can be done using free weights, resistance bands, or machine weights.  Aim to perform 2-3 sets of 8-12 repetitions of each exercise, with at least one day of rest in between strength training sessions.  Make sure to eat enough protein to support muscle building. Protein is important for muscle repair and growth, and it can help preserve muscle mass during weight loss.  Stay hydrated and get enough rest to allow your muscles time to recover and grow.  Be consistent with your exercise and nutrition plan and track your progress to help you stay motivated and see results.  Remember to always consult with a healthcare provider before starting any exercise program. | Similar | 1 | 1 | N/A |
| **Other (5)** | | | | | |
| When can you get pregnant after weight loss surgery? | It is generally recommended to wait at least 18 to 24 months after weight loss surgery before trying to become pregnant. This is because the body needs time to stabilize and recover after the surgery, and rapid weight loss can put a strain on the body and affect the nutritional status of the mother and developing fetus. It is also important for the mother to be able to maintain a healthy weight and nutritional status during pregnancy, which may be more challenging if she has recently undergone weight loss surgery. It is important to discuss your plans to become pregnant with your healthcare provider and to carefully consider the potential risks and benefits of pregnancy after weight loss surgery. Your healthcare provider can help you determine the best timing for pregnancy based on your individual circumstances and the type of weight loss surgery you have undergone. | Yes | 1 | 1 | N/A |
| Will the baby be healthy if the mother gets weight loss surgery while pregnant? | It is generally not recommended for women to have weight loss surgery while they are pregnant. Pregnancy is a time when proper nutrition and weight gain are important for the health of both the mother and the baby. Weight loss surgery can cause significant changes to the digestive system and may interfere with the absorption of nutrients, which can potentially impact the health of the baby. It is important for women who are considering weight loss surgery to discuss their options with their healthcare provider and to carefully weigh the potential risks and benefits. If a woman is considering weight loss surgery after pregnancy, it is typically recommended to wait at least six months to a year after giving birth to allow the body to fully recover. In the meantime, it is important for pregnant women and new mothers to focus on eating a healthy diet, getting regular exercise, and managing stress to support the health of themselves and their baby. | Similar | 1 | 3 | 3 |
| Is it faster to get bariatric surgery without going through insurance? | The process of getting weight loss surgery, also known as bariatric surgery, can vary depending on your insurance coverage and other factors. In some cases, going through insurance may result in a longer wait time for surgery, while in other cases, it may be faster.  If you have insurance coverage for weight loss surgery, the process may involve obtaining pre-authorization from your insurance company and meeting certain requirements, such as participating in a weight loss program or getting a referral from your primary care provider. These steps can add time to the process of getting surgery.  On the other hand, if you pay for the surgery out of pocket, you may be able to bypass some of these steps and potentially have the surgery more quickly. However, it is important to note that weight loss surgery is a major procedure and should not be rushed. It is important to carefully consider all of your options and to work with a qualified healthcare team to ensure a safe and successful outcome. | Similar | 1 | 1 | N/A |
| Can a woman still get pregnant after weight loss surgery? | Yes, it is possible for a woman to get pregnant after weight loss surgery. Weight loss surgery, such as gastric bypass or sleeve gastrectomy, can lead to significant weight loss, which can improve fertility and increase the chances of pregnancy.  However, it is important to note that weight loss surgery is not a substitute for birth control. If you are sexually active and do not want to get pregnant, it is important to use an effective form of contraception, such as condoms, birth control pills, or an intrauterine device (IUD).  It is also important to remember that weight loss surgery is a major procedure and it is important to allow your body time to heal and recover before trying to get pregnant. It is generally recommended to wait at least 18-24 months after weight loss surgery before trying to get pregnant.  If you are considering weight loss surgery and have questions about fertility or pregnancy, it is important to talk to your healthcare provider. They can help you understand the potential risks and benefits of the surgery and recommend the best course of action for you. | Yes, it is possible for a woman to get pregnant after weight loss surgery. However, the risk of pregnancy complications may be higher for women who have had weight loss surgery. Some studies have suggested that women who have had bariatric surgery may be at an increased risk of preterm birth, low birth weight, and cesarean delivery. It is important for women who are considering pregnancy after weight loss surgery to speak with their doctor about their specific situation and any potential risks. They can provide guidance on how to optimize the health of both the mother and baby during pregnancy. | 1, 2 | 1 | 2, 1 |
| Is it possible to get bariatric surgery without health insurance? | It is possible to get bariatric surgery without health insurance, but it can be more challenging and may be more expensive. Many health insurance plans cover at least a portion of the cost of bariatric surgery, so if you have insurance, it is generally recommended to check with your insurance company to see if the procedure is covered.  If you do not have health insurance, you may still be able to get bariatric surgery, but you will likely have to pay for it out of pocket. The cost of bariatric surgery can vary significantly depending on a number of factors, including the type of procedure, the location of the surgery, and the surgeon's fees. Some bariatric surgeons and hospitals may offer financing options or payment plans to help make the surgery more affordable.  It is important to carefully consider the costs and benefits of bariatric surgery, and to weigh the potential risks and complications against the potential benefits. If you are considering bariatric surgery and do not have health insurance, it is a good idea to speak with a bariatric surgeon or a medical professional to discuss your options and to determine if the surgery is appropriate for you. | Similar | 1 | 1 | N/A |
| Is it okay to take an appetite suppressant after weight loss surgery? Are there any negative consequences of taking one? | It is generally not recommended to take appetite suppressants after weight loss surgery. Appetite suppressants can interfere with the normal functioning of your digestive system and may cause negative side effects. Weight loss surgery, such as gastric bypass surgery, is a major procedure that can significantly alter your body's ability to digest and absorb food. Taking appetite suppressants on top of this can further disrupt your body's normal functioning and may cause complications.  It is important to speak with your surgeon or a healthcare professional before starting any new medication or supplement, including appetite suppressants, after weight loss surgery. They will be able to advise you on the safety and potential risks of taking appetite suppressants and help you determine the best course of action for your specific situation. | Similar | 1 | 2 | 3 |
| Does weight loss surgery reduce cravings for sugar? | Weight loss surgery may help to reduce cravings for sugar and other unhealthy foods in some individuals. This is because weight loss surgery, such as gastric bypass surgery or gastric sleeve surgery, can alter the hormones and brain signals that control hunger and fullness. As a result, some people may find that they have fewer cravings for sugary or high-fat foods after weight loss surgery.  It is important to note, however, that weight loss surgery is just one tool to help with weight loss and maintenance, and making healthy lifestyle changes, including following a balanced diet and engaging in regular physical activity, is essential for long-term success.  If you are experiencing cravings for sugar or other unhealthy foods after weight loss surgery, it is a good idea to speak with a healthcare professional or a registered dietitian. They can help you develop strategies to manage cravings and make healthy food choices. | Similar | 1 | 1 | N/A |
| Is weight loss surgery covered by Medicaid? | Weight loss surgery may be covered by Medicaid, depending on the specific Medicaid program and the individual's circumstances. Medicaid is a joint federal and state program that provides health coverage to low-income individuals and families. Each state operates its own Medicaid program, and coverage may vary depending on the state.  In general, Medicaid programs are required to cover certain medically necessary treatments, including weight loss surgery, if they are deemed medically necessary and appropriate. However, coverage may vary depending on the specific Medicaid program and the individual's circumstances.  If you are considering weight loss surgery and have Medicaid coverage, it is a good idea to contact your Medicaid program to determine what is covered under your plan. You can find contact information for your state's Medicaid program on the Centers for Medicare and Medicaid Services (CMS) website. | Similar | 1 | 1 | N/A |
| Can hiatal hernias be repaired during weight loss surgery? | It is possible to repair a hiatal hernia during weight loss surgery, depending on the size and location of the hernia and the specific type of weight loss surgery being performed.  A hiatal hernia is a condition in which part of the stomach protrudes through an opening in the diaphragm, the muscle that separates the chest from the abdomen. Hiatal hernias are relatively common and can be small or large.  If a hiatal hernia is small and not causing symptoms, it may not require treatment. However, if a hiatal hernia is causing symptoms or is large, it may be repaired during weight loss surgery. This may be done through the same incisions as the weight loss surgery or through additional incisions.  It is important to discuss the potential risks and benefits of repairing a hiatal hernia during weight loss surgery with a healthcare professional. They will be able to assess your individual situation and help you determine the best course of action. | Similar | 1 | 1 | N/A |
| Should insulin be stopped after weight loss surgery? | It is generally not recommended to stop insulin after weight loss surgery without the guidance of a healthcare professional. Insulin is a hormone that helps regulate blood sugar levels and is often prescribed to individuals with diabetes to help manage the condition.  Weight loss surgery can often help to improve insulin resistance and blood sugar control in individuals with diabetes, and some people may be able to reduce or stop their insulin use after surgery. However, this will depend on the individual's specific situation and the recommendations of their healthcare team.  If you are taking insulin and are considering weight loss surgery, it is important to speak with a healthcare professional, such as a primary care doctor or a bariatric surgeon, to determine the best course of action. They will be able to assess your health and weight loss goals and help you determine if stopping insulin is a safe and appropriate option for you.  It is also important to remember that weight loss surgery is just one tool to help with weight loss and maintenance, and a healthy lifestyle including a balanced diet and regular physical activity is essential for long-term success. | Similar | 1 | 1 | N/A |
| How do you slow down weight loss after weight loss surgery? | There are several strategies that you can use to slow down weight loss after weight loss surgery:  Increase your food intake: If you are losing weight too quickly after surgery, you may need to increase your food intake. This can be done by eating more frequently, adding additional high-protein snacks between meals, or increasing the portion sizes of your meals.  Eat more calorie-dense foods: Incorporating more calorie-dense foods, such as nuts, avocado, and olive oil, into your diet can help increase your calorie intake and slow down weight loss.  Focus on protein: Getting sufficient protein is important after weight loss surgery to help with muscle mass maintenance and weight loss. Incorporating protein-rich foods, such as chicken, fish, tofu, and beans, into your diet can help increase your protein intake and slow down weight loss.  Increase physical activity: Incorporating more physical activity into your routine can help increase your calorie expenditure and slow down weight loss. This can include activities such as walking, swimming, cycling, or strength training.  It is important to speak with a healthcare professional before making any changes to your diet or activity level after weight loss surgery. They will be able to provide guidance and recommendations based on your individual needs and goals. | Similar | 1 | 2 | 3 |
| Which website has the most accurate information about weight loss surgery? | There are many websites that provide information about weight loss surgery, but it is important to be cautious about the accuracy of the information. Some websites may provide biased or inaccurate information, so it is important to verify the information from multiple sources. Some reliable sources of information about weight loss surgery include:  The American Society for Metabolic and Bariatric Surgery (ASMBS) The American College of Surgeons (ACS) The Centers for Disease Control and Prevention (CDC) The National Institute of Diabetes and Digestive and Kidney Diseases (NIDDK) It is also a good idea to speak with a healthcare provider or a bariatric surgeon for personalized information about weight loss surgery. They can provide information based on your specific medical history and needs. | There are many websites that provide information about weight loss surgery, but it is important to be aware that not all of them may have accurate or reliable information. Here are a few tips to help you find credible and accurate sources of information about weight loss surgery:  Look for websites that are affiliated with reputable organizations, such as hospitals, universities, or professional medical associations. These organizations often have strict guidelines for the accuracy and reliability of the information they publish.  Check the credentials of the authors of the information you are reading. Are they qualified healthcare professionals, such as doctors, nurses, or dietitians?  Look for websites that have a privacy policy and terms of use, which can be signs that the website is trustworthy.  Be wary of websites that make exaggerated or unrealistic claims, or that try to sell you a product or service.  It is always a good idea to consult with a healthcare professional, such as a doctor or a registered dietitian, before making any decisions about weight loss surgery or other medical treatments. They can provide you with personalized and accurate information based on your specific circumstances and needs. | 2, 2 | 1 | 2, 2 |
